# Stable carbamate pathway towards organic–inorganic hybrid perovskites and aromatic imines†

**DOI:** 10.1039/d0ra07814j

**Published:** 2020-10-15

**Authors:** Kyu Hyung Lee, Sun Joo Kim, Hee Sun Park, Byung Wook Lim, Byeongno Lee, Young Jun Park, Wonwoo Nam, Nam Hwi Hur

**Affiliations:** Department of Chemistry, Sogang University Seoul 04107 Korea nhhur@sogang.ac.kr; Department of Bioinspired Science, Department of Chemistry and Nano Science, Ewha Womans University Seoul 03760 Korea

## Abstract

Methyl ammonium methyl carbamate (MAC), formulated as CH_3_NH_3_^+^CH_3_NHCO_2_^−^, was synthesized by reacting liquid methylamine with supercritical CO_2_, and its structure was refined by single-crystal X-ray diffraction. MAC is a white crystalline salt and is as reactive as methylamine, and is a very efficient alternative to toxic methylamine. We were able to produce hybrid perovskite MAPbI_3_ (MA = methyl ammonium) by grinding MAC with PbI_2_ and I_2_ at room temperature, followed by storing the mixed powder. Moreover, this one-pot method is easily scalable for the large-scale synthesis of MAPbI_3_ in a small vessel. We have also investigated the reactivity of MAC towards aromatic aldehydes in the absence of solvent. The solventless reactions afforded imines as exclusive products with over 97% yield, which show higher selectivity than the methylamine-based synthesis. Complete conversions were typically accomplished within 3 h at 25 °C. The results of this study emphasize the importance of solid carbamates such as MAC to develop an environmentally friendly process for the synthesis of various amine-based materials on the industrial scale.

## Abstract 2

A stable solid carbamate (MAC) composed of CH_3_NH_3_^+^ and CH_3_NHCO_2_^−^ units exhibits high reactivity toward inorganic iodide and aromatic aldehyde.

## Graphical abstract

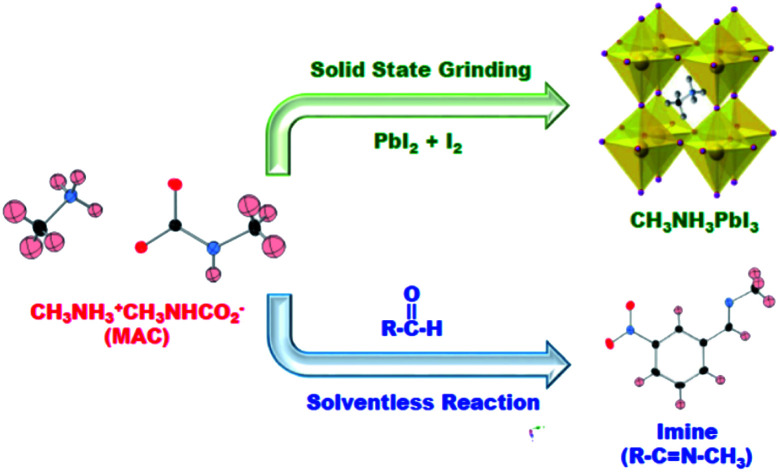

## Introduction

Methylamine is the simplest primary amine with the formula CH_3_NH_2_, and is widely used as a precursor for the synthesis of pharmaceuticals, pesticides, and organic–inorganic hybrid perovskites.^1–5^ It is a poisonous and intractable gas, which makes it challenging to handle. Accordingly, methylamine is used as a solution diluted with various solvents in the laboratory. However, the vapor of methylamine dissolved in a solution is still hazardous, which limits its extensive use. This disadvantage can be resolved by replacing methylamine with a solid carbamate salt that contains the methylamine moiety.

Primary amines (RNH_2_, R = alkyl), such as methylamine, are known for their strong interaction with carbon dioxide (CO_2_). Typically, two equivalents of RNH_2_ react with CO_2_ to form an alkyl ammonium carbamate salt (RNH_3_^+^RNHCO_2_^−^).^6–17^ The carbamate dissociates into the parent amines and CO_2_ by external stimuli. Due to this reversible interaction between amines and CO_2_, the solid carbamate was considered as a synthetic equivalent to liquid amine for the preparation of organic imines.^10–17^ Like other amines, the reaction of methylamine (CH_3_NH_2_) with CO_2_ can produce a methyl ammonium methyl carbamate (MAC, CH_3_NH_3_^+^CH_3_NHCO_2_^−^) salt. A couple of groups reported computational and experimental studies on the interaction between CH_3_NH_2_ and CO_2_,^18,19^ but isolation and structural characterization of the MAC had not been reported yet. For the first time, we isolated the MAC from the supercritical CO_2_ condition as a crystalline solid. The structure of MAC, determined using single-crystal X-ray diffraction, is composed of the cation (CH_3_NH_3_^+^) and anion (CH_3_NHCO_2_^−^) units, which are connected by weak inter-ionic hydrogen bonds. MAC has shown excellent reactivity towards inorganic compounds and organic substrates.

The organic–inorganic hybrid perovskites, such as methyl ammonium lead iodide (MAPbI_3_), were typically synthesized from the reaction between lead iodide (PbI_2_) and methyl ammonium iodide (MAI) in the presence of organic solvents.^2–5^ In a solution-based process, the low solubility of PbI_2_ interferes with the precise control of the desired stoichiometry and the large scale production of perovskite powders. Recently, several groups reported mechanically induced solid-state reaction methods for the synthesis of MAPbI_3_ from MAI and PbI_2_ precursors.^20–22^ However, high-speed ball milling is required to produce the perovskite MAPbI_3_ because of the low reactivity of the precursors. Reported herein is the superior reactivity of MAC towards PbI_2_ and iodine (I_2_) for the preparation of MAPbI_3_, which is accomplished in the absence of solvents. The solid-state reaction relies on the use of MAC, which facilitates the rapid and selective formation of MAPbI_3_ at ambient conditions. This solid-state process involves the evolution of CO_2_ gas, which leads to a forward reaction in the direction of perovskite formation. The release of methylamine enables us to synthesize the MAPbI_3_ powder by grinding the mixture alone, followed by storing the mixed powder at the desired temperature. We also report our studies of the solid-state reactivity of MAC toward aromatic aldehydes for the synthesis of aromatic imines.^23,24^

The solvent-free reactions were performed as a one-pot process, producing the imine derivatives as exclusive products in over 97% yields at ambient conditions. This novel method using MAC allows us to avoid the use of volatile and toxic methylamine. It provides a novel sustainable approach for the synthesis of inorganic materials as well as organic compounds.

## Experimental

### Chemicals

All the chemicals were obtained from commercial suppliers and were used without further purification. Lead iodide (99.99%, PbI_2_) and iodine (≥99.99%, I_2_) were purchased from Alfa Aesar. *N*,*N*-Dimethyl formamide (DMF) and hydroiodic acid (55% w/w, HI) were purchased from Fisher Scientific. Benzaldehyde, 2-methoxybenzaldehyde, 4-chlorobenzaldehyde, 4-bromobenzaldehyde, 3-nitrobenzaldehyde, cinnamaldehyde, 2-methoxycinnamaldehyde, salicylaldehyde, 2-nitrocinnamaldehyde, benzophenone, and methylamine solution (33 wt% in ethanol) were purchased from Sigma-Aldrich.

### Synthesis of methyl ammonium methyl carbamate (MAC)

The reaction was conducted in a stainless steel autoclave reactor with a glass liner (Parr 4760, 300 mL). A methylamine solution (33 wt% in ethanol, 100.0 mmol) was introduced into a glass liner charged with dry ice (50 g). After attaching the gauge and gauge block assembly, the autoclave was placed in an oil bath on a hot plate and was heated at 80 °C. The CO_2_ pressure was maintained in the range of 90 to 110 bar. After reacting for 10 h, the autoclave was removed from the oil bath and was then cooled to room temperature. The white crystalline powders formed in the glass liner were filtered in air, followed by washing with cold methanol, cold diethyl ether, and pentane. The resulting solid was dried under vacuum for 5 h, which was confirmed as a methyl ammonium methyl carbamate (MAC) salt. The yield of MAC based on the methylamine used was 95.2%.

**Precaution**: MAC could be harmful for health due to its sublimation character. The equipment of adequate ventilation is highly recommended for handling the MAC to avoid vapor inhalation.

### Synthesis of methyl ammonium lead iodide (MAPbI_3_)

A typical procedure is as follows: lead iodide (0.461 g, 10 mmol) and iodine (0.254 g, 10 mmol) were mixed with MAC (0.106 g, 5.0 mmol), which was then ground using a pestle and mortar in an argon-filled glove box. The ground powder was stored in a vial (10 mL) placed in a Parr reactor and allowed to react at room temperature for 16 h. The resulting product was analyzed by X-ray diffraction and solid UV spectroscopy. The yield of MAPbI_3_ based on the MAC used was over 97.2%. In a similar manner, we prepared MAPbI_3_ samples by storing the ground powder at 50 and 100 °C for 1 h, respectively.

### Fabrication of perovskite MAPbI_3_ films

Perovskite MAPbI_3_ films were fabricated according to the spin coating method previously reported.^2–5,25^ In short, a solution of HPbI_3_ was prepared by dissolving PbI_2_ (1.0 mmol) in an acidified solution of DMF (1 mL) with hydroiodic acid (1.0 mmol), followed by stirring for 1 h. The resulting solution was used as the precursor solution. The HPbI_3_ solution was deposited on the FTO/TiO_2_ substrate with a size of 2 × 2.5 cm^2^ by spin-coating at 4000 rpm for 20 s. The HPbI_3_ film was dried on a hotplate at 100 °C for 30 min. The dried film was stored in a vial and then kept in an autoclave reactor filled with 0.16 g of MAC. Finally, heating the reactor at 100 °C for 2 h yielded perovskite MAPbI_3_ film. The thickness of the perovskite film was controlled by the HPbI_3_ concentration.

### General procedure for the preparation of imines from the solventless reactions between MAC and aromatic aldehydes

A typical procedure is as follows: a round-bottom flask was charged with 0.54 g of MAC (5.0 mmol) and an aromatic aldehyde (10.0 mmol) under the nitrogen atmosphere. The mixture was stirred at ambient temperature until the reaction was completed. The reaction progress was carefully monitored using ^1^H NMR spectroscopy. Complete conversion to imine typically took about 3 h at room temperature. No by-product, besides the imine, was identified from the reaction mixture. The yields of immines based on the MAC used were typically over 97%. The products were characterized by ^1^H and ^13^C NMR spectroscopy.

### Growth of 3-nitrobenzylidene methanamine single crystals

A solution was prepared by dissolving 3-nitrobenzylidene methanamine (0.1 g) in a 5 mL of pentane. The solution was stored in a tightly locked bottle for 2 days. White crystals of 3-nitrobenzylidene methanamine were slowly grown from the solution, which were recovered by vacuum filtration.

### Characterization

Powder X-ray diffraction (XRD) measurements were performed on a Rigaku DMAX 2500 diffractometer (Rigaku, Japan) with Cu Kα radiation (*λ* = 1.5406 Å) operated at 40 kV and 150 mA. The UV-visible absorption spectra were recorded using a Lambda 950 spectrophotometer (PerkinElmer, USA). High-resolution scanning electron microscopy analyses were carried out using a Hitachi S-5500 microscope (Hitachi, Japan). The operating acceleration voltage is 15 kV. Thermogravimetric analysis was carried out using a TGA 2050 instrument (TA Instruments, USA). The sample was placed on a platinum pan for each run and analyzed in air or oxygen from 25 to 900 °C at a heating rate of 5 °C min^−1^. GC/MS data were recorded on Agilent 5973N, and elemental analyses were obtained using a Carlo Erba EA1180 at the Organic Chemistry Research Center at Sogang University. ^1^H NMR and ^13^C NMR spectra in solution were recorded on a Varian 400 MHz Gemini operating at 400 MHz for ^1^H and 100 MHz for ^13^C, respectively. All chemical shifts were referenced to tetramethylsilane. Single-crystal X-ray diffraction data were collected using a Bruker SMART AXS diffractometer equipped with a monochromator with a Mo Kα (*λ* = 0.71073 Å) incident beam.

## Results and discussion

### Structural characterization of MAC

**Fig. 1:**
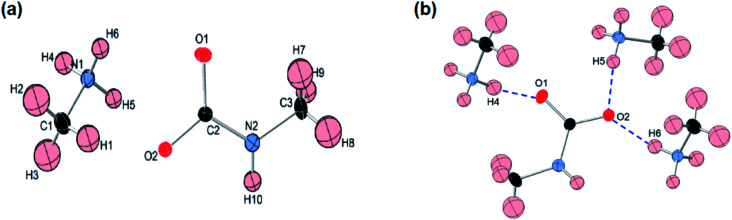
(a) ORTEP drawing and numbering scheme for the methyl ammonium cation and methyl carbamate anion in the formula unit of the CH_3_NH_3_^+^CH_3_NHCO_2_^−^ salt. All the atoms are shown as 30% probability displacement ellipsoids. Covalent bonds are linked by solid lines. Red, black, blue, and pale pink ellipsoids represent O, C, N, and H atoms, respectively. (b) Illustration of hydrogen bonds in MAC. Blue dashed lines represent the hydrogen bonds in which the O⋯H distances are shorter than 2.0 Å. For clarity, other O⋯H distances longer than 2.0 Å are omitted.

Like most primary amines, methylamine is known to react readily with carbon dioxide to yield methyl ammonium methyl carbamate (MAC). MAC is known, but its crystal structure has not been determined. Single crystals of MAC were grown from a methylamine solution charged with pressurized CO_2_ gas. After the reaction was completed, the reactor was cooled very slowly down to 0 °C. The solid-state structure of MAC was determined by single-crystal X-ray diffraction. As illustrated in Fig. 1(a), the MAC is composed of the cation (CH_3_NH_3_^+^) and anion (CH_3_NHCO_2_^−^) parts, which is structurally analogous to typical carbamate salts.

There are three different C–N bonds of MAC: C1–N1, C2–N2, and C3–N2. The C1–N1 bond distance of 1.479 Å in the methyl ammonium cation closely matches the C–N bond length of free CH_3_NH_2_ (1.471 Å).^18^ The C3–N2 bond (1.452 Å) in the anion is slightly shorter than the C1–N1 one. The C2–N2 bond (1.359 Å) connected to the CO_2_ group is much shorter than the C1–N1 bond, indicating than the CO_2_ group is firmly bound to the CH_3_NH_2_ moiety. The two C–O bond distances are virtually identical. The bond lengths of C2–O1 and C2–O2 are 1.269 and 1.279 Å, respectively.

**Fig. 2:**
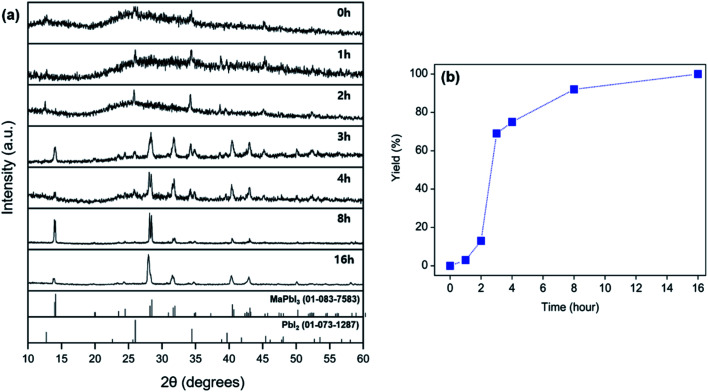
(a) Powder XRD patterns of products obtained from grinding a mixture of MAC, PbI_2_, and I_2_ at intervals of 1, 2, 4, 8, and 16 h at 25 °C. Theoretical diffraction data of MAPbI_3_ and PbI_2_ are provided for reference at the bottom of the XRD patterns. (b) Yields of MAPbI_3_ as a function of time for the solid-state reactions performed at 25 °C.

A notable feature is that there are strong inter-ionic interactions ascribed to hydrogen bonds between the cation and anion units. Each methyl carbamate anion is hydrogen-bonded to three neighbouring methyl ammonium cations, which are illustrated in Fig. 2(b). The three O⋯H distances of O1–H4, O2–H5, and O2–H6 are 1.838, 1.839, and 1.908 Å, respectively. On the other hand, the H atom attached to the NH group of the anion weakly interacts with the O atoms. Their bond distances are much longer than those of the three O–H bonds. The stable network structure of MAC in the solid-state is due presumably to the inter-ionic hydrogen bonds between the cation and anion species. Detailed crystallographic data are presented in Tables S1 and S2 in the ESI.†

### Synthesis of MAPbI_3_ by solid-state grinding

To explore the reactivity of MAC towards inorganic substances, the reaction among MAC, PbI_2_, and I_2_ was investigated to make perovskite MAPbI_3_. Specifically, the precursors were ground using a mortar and pestle, and the mixed powder was allowed to react at 25 °C in the absence of intermediate grindings. A facile color change from white to black was noticed in the ground powder, implying that the conversion of precursors to MAPbI_3_ occurs *via* the mechanically induced solid-state reaction. The products were carefully analyzed by powder XRD data as a function of storage time at 25 °C, which is illustrated in Fig. 2(a). Peak intensities of MAPbI_3_ increased with increasing storage time. The XRD pattern collected after storing the powder for 4 h clearly shows new peaks corresponding to the MAPbI_3_ phase. On the other hand, intensities of main PbI_2_ peaks are significantly suppressed after the 4 h reaction time. Complete conversion was achieved in a 16 h period and only MAPbI_3_ was obtained from the solid-state reactions. It is thus evident that MAC is a very effective reagent for the synthesis of MAPbI_3_ through the solid-state reaction alone. The yield of MAPbI_3_ was estimated by the intensity value of (220) peak at 28.45° in MAPbI_3_ relative to (011) peak at 25.92° in unreacted PbI_2_. Fig. 2(b) shows yields of MAPbI_3_ as a function of reaction time, clearly demonstrating that yield increases with increasing reaction time.

**Fig. 3:**
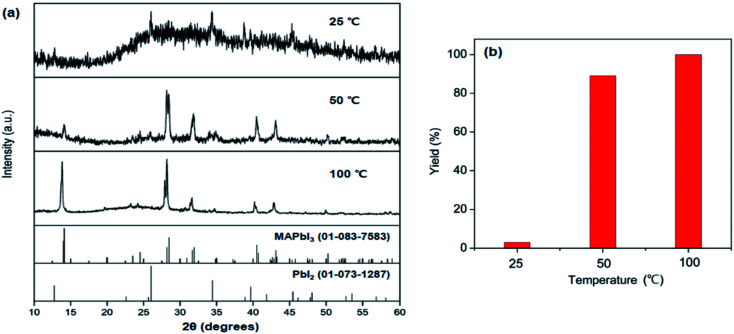
(a) Powder XRD patterns of the products synthesized in solid-state reactions of MAC, PbI_2_, and I_2_ at 25 °C, 50 °C, and 100 °C for 1 h. (b) Yields of MAPbI_3_ obtained from the three reactions.

To evaluate the reaction rate at elevated temperature, ground powders were allowed to react in a vial at 50 and 100 °C for 1 h, without any additional agitation. Powder XRD patterns of the products in Fig. 3 reveal that the formation of MAPbI_3_ is significantly accelerated at high temperatures. Remarkably, the complete conversion was accomplished within 1 h at 100 °C. All the peaks from the product at 100 °C are well matched with those of MAPbI_3_, and no PbI_2_ peaks are visible. These time- and temperature-dependent results suggest that the solid-state pathway using MAC not only provides high reactivity but also renders increased selectivity towards the formation of MAPbI_3_ compared to solution-based methods. In contrast, most solution methods involve a combination of well-soluble methyl iodide (CH_3_I) and less-soluble lead iodide (PbI_2_). This difference in solubility leads to the formation of MAPbI_3_ along with unreacted PbI_2_.

### Fabrication of MAPbI_3_ film

**Fig. 4:**
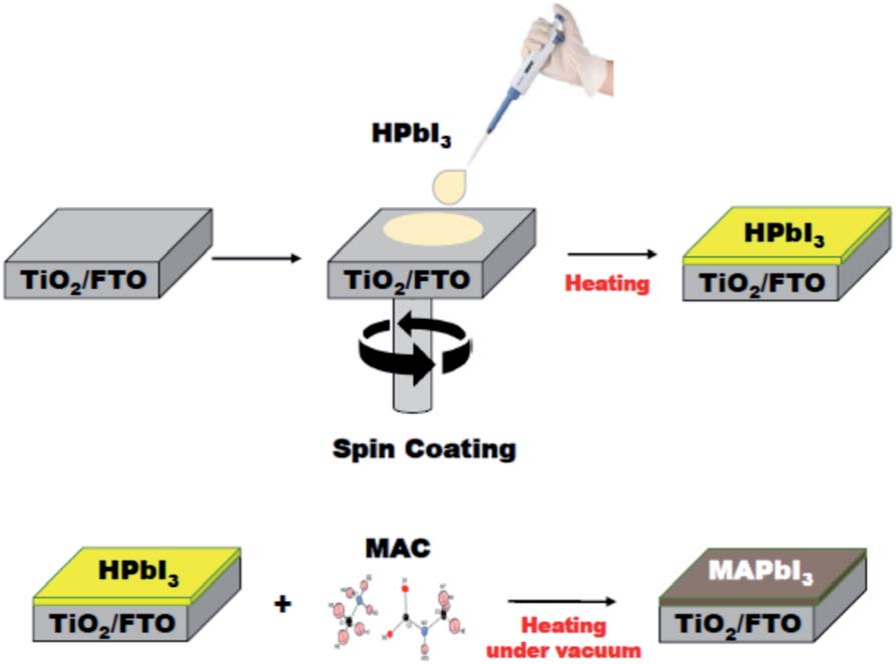
Schematic illustration of the formation of MAPbI_3_ film. Fabrication of HPbI_3_ film on a TiO_2_/FTO substrate was achieved by the spin-coating method (top panel). Conversion of HPbI_3_ film to MAPbI_3_ film using MAC, where the vapor-phase reaction was performed in an autoclave reactor filled with the MAC powder (bottom panel).

The solid-state reaction route can be extended to the fabrication of MAPbI_3_ film applicable for perovskite solar cells and modules. The MAPbI_3_ film was grown on a TiO_2_/FTO substrate using the MAC as a source of methylamine. As shown in Fig. 4, the HPbI_3_ film was first deposited by a spin-coating method. An important feature is that the HPbI_3_ solution can be easily prepared by adding hydroiodic acid (HI) into PbI_2_. Moreover, the solution is well miscible with various solvents, which is advantageous for the production of high-quality films without agglomerates. The deposited film was placed in an autoclave reactor filled with the MAC powder. Heating the reactor at 100 °C leads to the facile dissociation of MAC into methylamine and CO_2_. The methylamine gas released spreads evenly over the HPbI_3_ film, which can accelerate the conversion of HPbI_3_ to MAPbI_3_. In the present process, the film thickness relies on the concentration of HPbI_3_, which was controlled from 500 nm to 1000 nm. The MAC precursor enables the formation of highly uniform perovskite MAPbI_3_ films through the vapor to solid reaction. Scanning electron microscopy (SEM) was employed to examine the surface morphology of the MAPbI_3_ films. As shown in the top-view SEM image (Fig. S1†), the MAPbI_3_ film has a dense surface morphology without pin-holes and appears to mostly consist of grains larger than 1 μm.

**Fig. 5:**
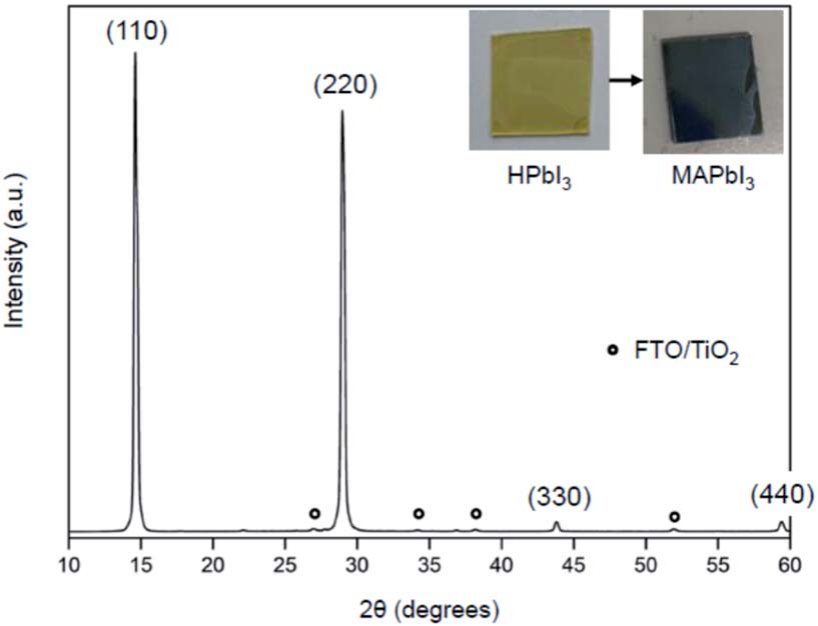
XRD patterns of the MAPbI_3_ film converted from the HPbI_3_ film *via* vapor-phase reaction using MAC. Asterisk marks indicate diffraction peaks of TiO_2_/FTO. The inset shows photographs of HPbI_3_ (left) and MAPbI_3_ (right) films. The images were taken before (left) and after (right) the reaction between HPbI_3_ and MAC.

XRD measurements characterized the phase formation and crystallinity of MAPbI_3_ films prepared by the present method using MAC. Two sharp and intense peaks appeared at 14.23°, and 28.45° are indexed to (110) and (220) reflections of MAPbI_3_, respectively (see Fig. 5). The full-width at half-maximum (FWHM) in the (110) plane is 0.24°, which is about the same as those of typical MAPbI_3_ films prepared by conventional methods.^26,27^ The XRD data show not only a high degree of (110) type preferential grain growth but also excellent crystallinity of the MAPbI_3_ film. It is worth mentioning that high-quality perovskite films with smooth and pinhole-free films over specified areas can also be prepared by heat treatment of lead iodide precursors in the presence of the CH_3_NH_2_ gas.^28^ Methylamine gas has been shown to have a significant effect on recrystallization of the MAPbI_3_ material. This vapor-assisted annealing is a convenient technique for the fabrication of MAPbI_3_ films. However, a drawback is that it is difficult to control the amount of gas that requires complete conversion accurately. In contrast, MAC has the advantage of being easy to handle and being able to use accurately as needed.

The formation of MAPbI_3_ film on a TiO_2_/FTO substrate by the vapor-phase reaction with MAC was also evaluated by UV-visible spectroscopy, which is shown in Fig. S2.† The UV-visible spectrum of the MAPbI_3_ film shows an absorption edge at about 770 nm, which is the characteristic onset corresponding to the MAPbI_3_ phase. The onset of absorption cannot be determined accurately because the slope change is small near the absorption edge. However, the optical band gap, *E*_g_, can be determined from the extrapolation of the linear part of a Tauc plot, as shown in the inset of Fig. S2.† The bandgap is 1.57 eV, which is similar to that reported in other MAPbI_3_ films.^29,30^ The spectrum shows a gradual increase of absorbance in the range from 750 down to 350 nm, which is attributed to light scattering effects caused by the large MAPbI_3_ grains.

### Solvent-free synthesis of aromatic imines using MAC

To demonstrate the generality of the MAC molecule, we investigated the one-pot reactions of MAC with aromatic aldehydes. The addition of MAC to the liquid aromatic aldehyde in the absence of solvents yielded the corresponding imine as a sole product. A distinctive color change was noticed upon the addition of MAC. The solution was allowed to react in a vial without any agitation. The condensation reaction proceeded to form the C=N double bond with the simultaneous loss of water, which appears to proceed *via* the dissociation of MAC into methylamine and CO_2_. Nearly complete conversion of aromatic aldehyde to imine was typically achieved even at 25 °C within 3 h, where the product was carefully analyzed using ^1^H and ^13^C NMR spectroscopy. Neither by-products nor unreacted aromatic aldehydes were identified from the reaction mixture, indicating that the neat reaction is highly selective. Table 1 summarizes the results of the reactions between MAC and aromatic aldehydes along with detailed reaction conditions.

**Table 1:** Reactions of MAC with aromatic aldehydes^a^

Entry	Reactant	Product	Reaction time (h)	Yield^b^ (%)	Remark
1	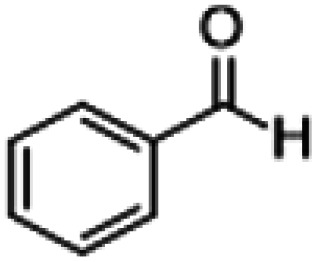	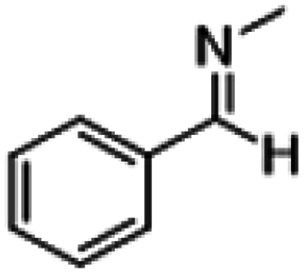	3	>99	Yellow liquid
2	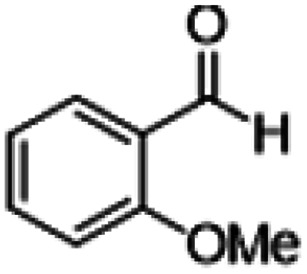	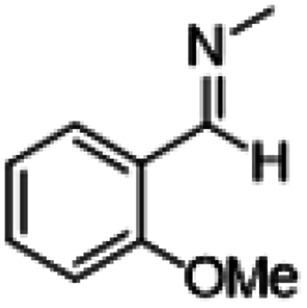	3	>99	Yellow liquid
3	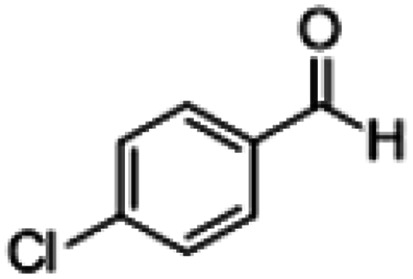	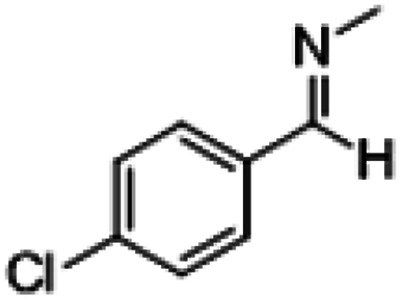	3	>99	Pale yellow liquid
4	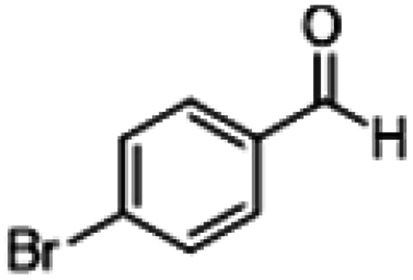	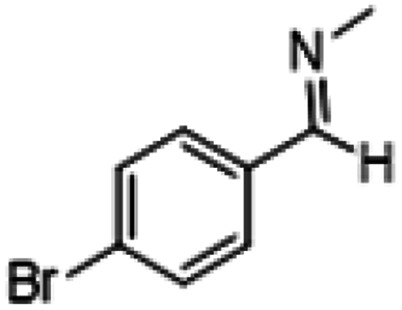	3	>99	Pale yellow liquid
5	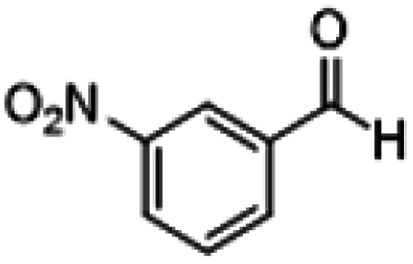	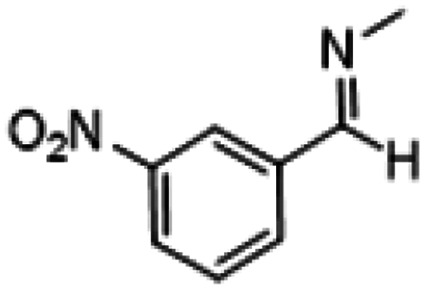	3	>97	Pale yellow liquid
6	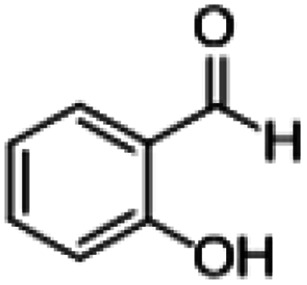	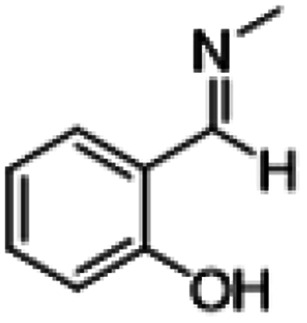	1	>99	Yellow liquid
7	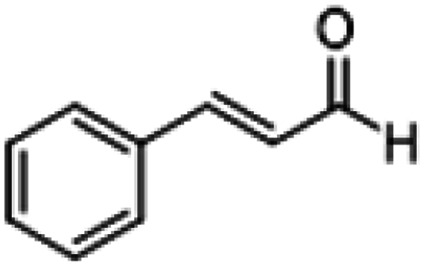	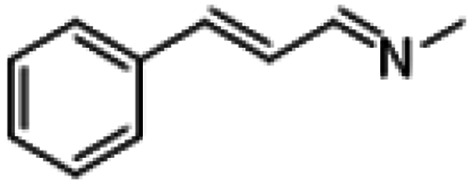	0.5	>96	Yellow liquid
8	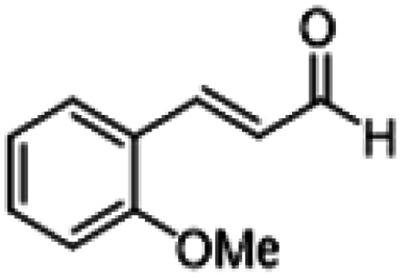	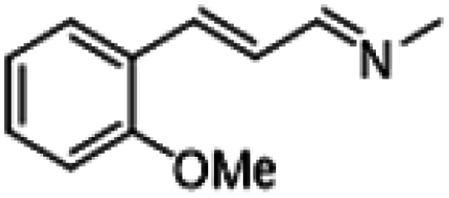	3	>99	Yellow liquid

a Reaction conditions: 1 (5 mmol, 0.53 g), carbonyl reactant 2 (10 mmol), no solvent, in a 10 mL vial at 25 °C.

b Isolated yield based on MAC was determined using ^1^H NMR.

For comparison, liquid methylamine dissolved in ethanol was reacted with cinnamaldehyde to determine whether the methylamine-based reaction also yields the imine selectively. The reaction was performed in ethanol under the same conditions, which produced not only the imine but also unknown impurities (Table 2, entry 1). The selectivity towards the imine was relatively low, which is presumably due to the presence of ethanol. When MAC was used as a methylamine source, on the other hand, the yield was well above 97%, and no by-products were identified by ^1^H NMR analysis. (see Table 2, entry 2). The neat reaction between MAC and cinnamaldehyde clearly shows the enhanced selectivity compared to the conventional methylamine-based method. The reason for the high selectivity is that methylamine and CO_2_ gases are rapidly released from MAC. Methylamine gas is anhydrous and highly reactive. This one-pot solvent-less synthesis of imine using MAC is unprecedented, which provides an environmentally benign approach for preparing various imines. Moreover, this reaction is easily scalable and can produce imine on a large scale in a small container without separation and purification steps. As an illustrative example, the same reaction was performed on a 50 mmol scale under identical conditions. Almost the same yield was obtained (see Table 2, entry 3).

**Table 2:** Reactions of cinnamaldehydes with MAC and methylamine in ethanol (33 wt%)^a^

Entry	Methyl amine (mmol)	Product	Yield (%)	Remark
1	CH_3_NH_2_ in ethanol (5.0 mmol)	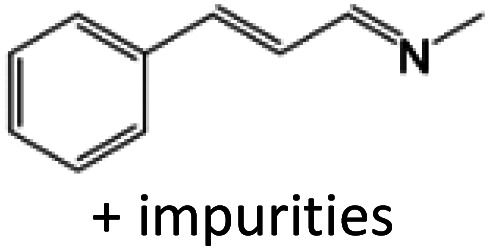	<84^b^	c
2	MAC (5.0 mmol)	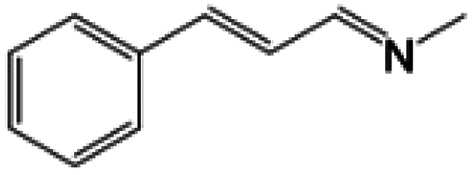	>97^b^	Yellow liquid
3	MAC (50.0 mmol)	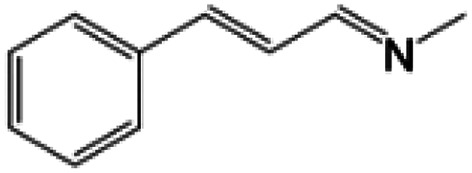	>97^c^	Yellow liquid

a Reaction conditions: cinnamaldehyde/methyl amine = 2/1, no solvent, the precursors were reacted at 25 °C for 0.5 h.

b Isolated yield based on MAC was determined using ^1^H NMR.

c Product(s) resulting from the reaction(s) at the olefin group in cinnamaldehyde were identified by ^1^H NMR.

**Fig. 6:**
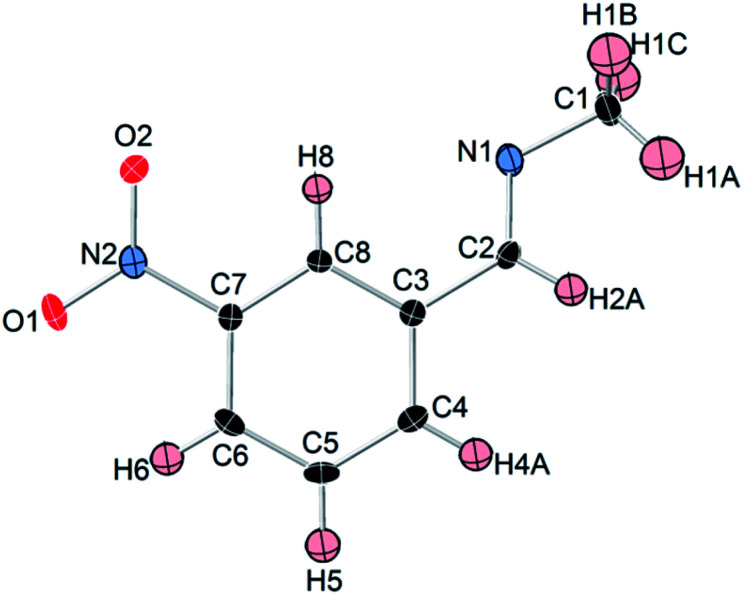
ORTEP drawing and numbering scheme of 3-nitrobenzylidene methanamine. Non-hydrogen atoms are shown as 30% probability displacement ellipsoids. Red, black, blue, and pale pink ellipsoids represent O, C, N, and H atoms, respectively.

To convince the formation of an imine (–C=N–) bond, we grew single crystals of 3-nitrobenzylidene methanamine by the recrystallization of the initial yellow product in pentane. Its solid-state structure was determined by single-crystal X-ray diffraction. As illustrated in Fig. 6, the molecular structure is composed of the methylamine and aromatic aldehyde units linked by a N=C double bond. The C2–N1 bond length (1.263 Å) of the molecule is considerably shorter than that of free methylamine, which is an intermediate between single (C–N) and triple (C≡N) bonds. It is quite similar to those of typical C=N double bonds.^31–34^ Accordingly, the formation of imine group is evident. Details of the crystal structure of 3-nitrobenzylidene methanamine including bond lengths and angles are given in Tables S3 and S4 in the ESI.†

## Conclusion

We have demonstrated the excellent reactivity of a newly synthesized methyl ammonium methyl carbamate (MAC) solid towards inorganic compounds for the preparation of hybrid perovskite MAPbI_3_ and also organic compounds for the synthesis of imines. MAC turned out to be a highly effective alternative to toxic methylamine. A remarkable benefit of MAC is that it enables us to use the precise amount of methylamine as well as it can be used safely in a laboratory. This one-pot solvent-less process provides an environmentally benign synthesis of various methylamine-based materials under relatively mild conditions, which surpasses gas-based or liquid-based methods in environmental and economic aspects. Also, this approach renders the scalable production of methylamine-based materials on a large scale.

## Conflicts of interest

There are no conflicts to declare.

## Supplementary Material

RA-010-D0RA07814J-s001

RA-010-D0RA07814J-s002
