# Supplementary material for: Stable carbamate pathway towards organic–inorganic hybrid perovskites and aromatic imines
Source: RSC Adv. 2020 Oct 15;10(62):38055–62. doi: 10.1039/d0ra07814j (PMC9057179; doi:10.1039/d0ra07814j)
Supplement: RA-010-D0RA07814J-s001 [file RA-010-D0RA07814J-s001.pdf]

Electronic Supplementary Material (ESI) for RSC Advances.
This journal is © The Royal Society of Chemistry 2020

Supporting Information

Solid carbamate pathway towards organic-inorganic hybrid perovskites and aromatic imines

Kyu Hyung Lee,[a] Sun Joo Kim,[a] Hee Sun Park,[a] Byung Wook Lim,[a] Byeongno Lee,[a] Young Jun Park,[b] Wonwoo Nam,[b] Nam Hwi Hur [a,*]

[a] Department of Chemistry, Sogang University, Seoul 04107, Korea

[b] Department of Bioinspired Science, Department of Chemistry and Nano Science, Ewha Womans University, Seoul 03760, Korea

*E-mail: nhhur@sogang.ac.kr

X-ray crystallography. A single crystal of methyl ammonium methyl carbamate (MAC) was selected by a nylon loop (Hampton Research Co.) placed on a handmade cooper plate, which was placed inside a liquid N_2 Dewar vessel at approximately -40 ºC and was mounted on a goniometer head in a N_2 cryostream. Data collections were carried out in a Bruker SMART AXS diffractometer equipped with a monochromator with a Mo Kα (λ = 0.71073 Å) incident beam. The charge-coupled device (CCD) data were integrated and scaled using the Bruker-SAINT software package, and the structure was solved and refined using SHEXTL V 6.12.[1] Hydrogen atoms were located in the calculated

positions. The crystal data for MAC: $C_3H_{10}N_2O_2$, Orthorhombic, *Pna2₁*, Z = 4, a = 9.3493(2), b = 6.9787(1) , c = 9.1550(2) Å, $\alpha=\beta=\gamma$ 90°, V = 597.33(2) Å^3, μ = 0.097 mm^{-1}, ρ_{calcd} = 1.180 g/cm^3, R_1 = 0.0243, and wR_2 = 0.0589 for 1,444 unique reflections and 104 variables. The crystallographic data for MAC are listed in Table S1, and Table S2 lists the selected bond distances and angles. CCDC-2023499 for MAC contains the supplementary crystallographic data for this paper. These data can be obtained free of charge via www.ccdc.cam.ac.uk/data_request/cif (or from the Cambridge Crystallographic Data Centre, 12, Union Road, Cambridge CB2 1EZ, UK; fax: (+44) 1223-336-033; or deposit@ccdc.cam.ac.uk).

Table S1. Crystal data for methyl ammonium methyl carbamate ($CH_3NH_3^+CH_3NHCO_2^-$).

Empirical formula	$C_3H_{10}N_2O_2$
Formula weight	106.13
Temperature (K)	100(2)
Wavelength (Å)	0.71073
Crystal system/space group	Orthorhombic, $Pna2_1$
Unit cell dimensions	
a (Å)	9.3493(2)
b (Å)	6.9787(1)
c (Å)	9.1550(2)
α (°)	90
β (°)	90
γ (°)	90
Volume (Å^3)	597.33(2)
Z	4
Calculated density (g/cm^{-3})	1.180
Absorption coefficient (mm^{-1})	0.097
Reflections collected	12394
Independent reflections [R(int)]	1436 [0.0243]
Refinement method	Full-matrix least-squares on F^2
Data/restraints/parameters	1436/1/104
Goodness-of-fit on F^2	1.190

Table S2. Selected bond distances (Å) and bond angles (°) for methyl ammonium methyl carbamate ($CH_3NH_3^+CH_3NHCO_2^-$).

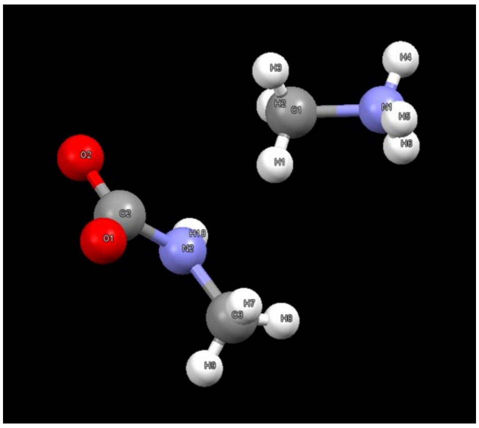

Number	Object1	Object2	Length
1	N1	H5	0.91(1)
2	N1	H4	0.91(1)
3	N1	H6	0.84(1)
4	N1	C1	1.479(1)
5	C1	H3	0.93(2)
6	C1	H2	0.98(2)
7	C1	H1	0.94(1)
8	C3	H8	0.89(2)
9	C3	H7	0.98(2)
10	C3	H9	0.95(1)
11	C3	N2	1.452(1)
12	N2	H10	0.83(1)
13	N2	C2	1.3592(9)
14	C2	O2	1.2787(9)
15	C2	O1	1.2694(8)

Number	Atom1	Atom2	Atom3	Angle
1	C1	N1	H4	110.4(8)
2	C1	N1	H5	109.4(7)
3	C1	N1	H6	109.2(8)
4	H4	N1	H5	109(1)

5	H4	N1	H6	109(1)
6	H5	N1	H6	110(1)
7	N1	C1	H2	107.8(9)
8	N1	C1	H3	109(1)
9	N1	C1	H1	107.5(8)
10	H2	C1	H3	111(1)
11	H2	C1	H1	109(1)
12	H3	C1	H1	112(1)
13	C3	N2	C2	122.93(6)
14	C3	N2	H10	119.3(8)
15	C2	N2	H10	117.4(8)
16	N2	C3	H9	111.7(7)
17	N2	C3	H8	112(1)
18	N2	C3	H7	109(1)
19	H9	C3	H8	110(1)
20	H9	C3	H7	109(1)
21	H8	C3	H7	105(2)
22	N2	C2	O2	117.53(6)
23	N2	C2	O1	119.47(6)
24	O2	C2	O1	122.99(6)

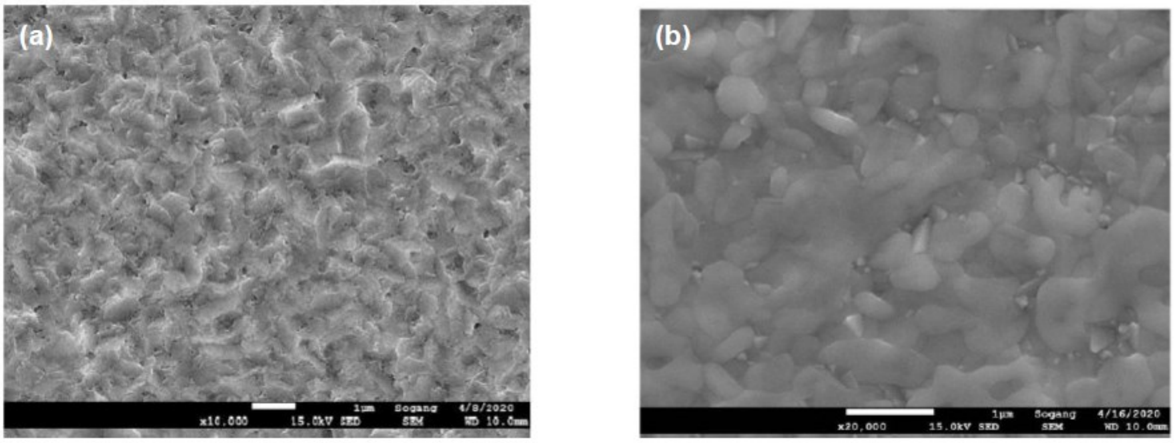

Figure S1. Top-view SEM image of the $MAPbI_3$ layer on a TiO_2/FTO substrate (a) before and (b) after reaction with MAC vapors.

X-ray crystallography. A single crystal of 3-nitrobenzylidene methanamine was selected by a nylon loop (Hampton Research Co.) placed on a handmade cooper plate, which was placed inside a liquid N_2 Dewar vessel at approximately -40 ºC and was mounted on a goniometer head in a N_2 cryostream. Data collections were carried out in a Bruker SMART AXS diffractometer equipped with a monochromator with a Mo Kα (λ = 0.71073 Å) incident beam. The charge-coupled device (CCD) data were integrated and scaled using the Bruker-SAINT software package, and the structure was solved and refined using SHEXTL V 6.12.[1] Hydrogen atoms were located in the calculated positions. The crystal data for 3-nitrobenzylidene methanamine: $C_8H_8N_2O_2$, Monoclinic, $P12_1/c1$, Z = 8, a = 7.4527(2), b = 18.3181(4) , c = 11.3999(2) Å, α=90, β= 90.869, γ 90°, V = 1556.13 (6) $Å^3$, μ = 0.103 mm^{-1}, ρ_{calcd} = 1.401 g/cm^3, R_1 = 0.0379, and wR_2 = 0.1055 for 3852 unique reflections and 219 variables. The crystallographic data for methyl 3-nitrobenzylidene methanamine are listed in Table S3, and Table S4 lists the selected bond distances and angles. CCDC-2023500 for 3-nitrobenzylidene methanamine contains the supplementary crystallographic data for this paper. These data can be obtained free of

charge via www.ccdc.cam.ac.uk/data_request/cif (or from the Cambridge Crystallographic Data Centre, 12, Union Road, Cambridge CB2 1EZ, UK; fax: (+44) 1223-336-033; or deposit@ccdc.cam.ac.uk).

Table S3. Crystal data for 3-nitrobenzylidene methanamine.

Empirical formula	$C_8H_8N_2O_2$
Formula weight	164.16
Temperature (K)	100(2)
Wavelength (Å)	0.71073
Crystal system/space group	Monoclinic, *P1 2₁/c1*
Unit cell dimensions	
a (Å)	7.4527(2)
b (Å)	18.3181(4)
c (Å)	11.3999(2)
α (°)	90
β (°)	90.869 (1)
γ (°)	90
Volume (Å^3)	1556.13(6)
Z	8
Calculated density (g/cm^{-3})	1.401
Absorption coefficient (mm^{-1})	0.103
Reflections collected	12394
Independent reflections [*R*(int)]	3091 [0.0379]
Refinement method	Full-matrix least-squares on F^2
Data/restraints/parameters	3091/1/219
Goodness-of-fit on F^2	1.014

Table S4. Selected bond distances (Å) and bond angles (°) for 3-nitrobenzylidene methanamine.

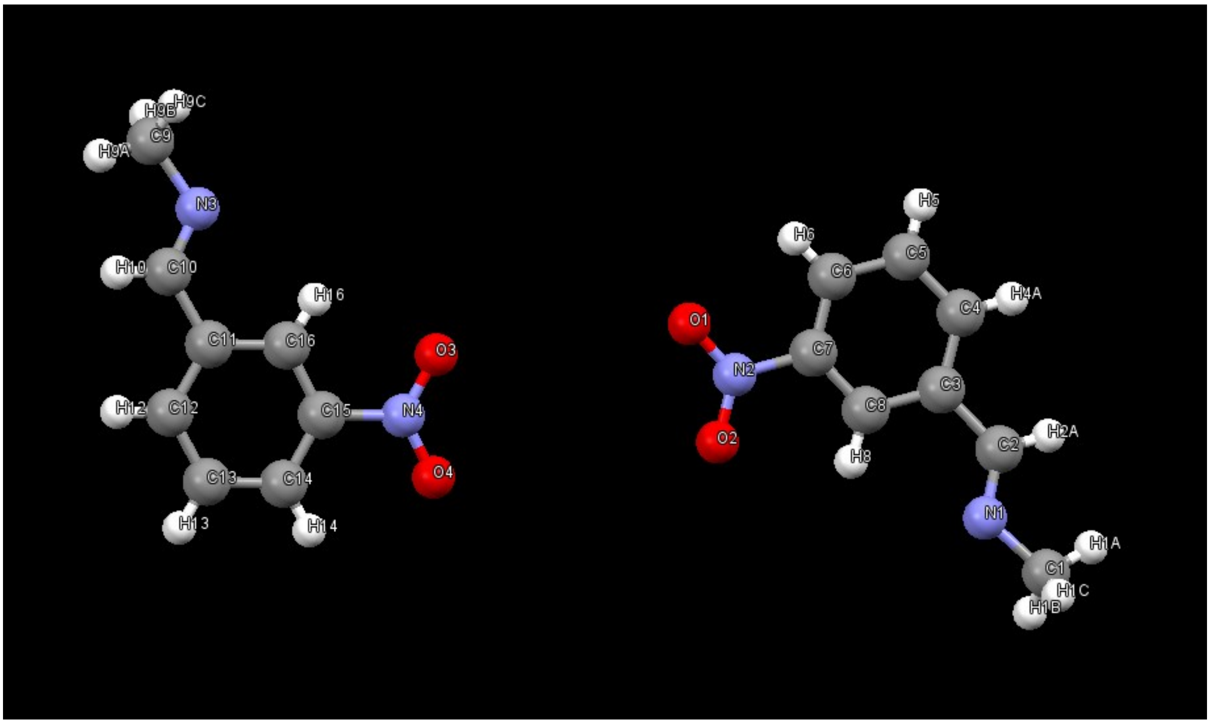

Number	Object1	Object2	Length
1	N2	O2	1.227(1)
2	N2	O1	1.233(1)
3	N2	C7	1.468(1)
4	C7	C6	1.387(2)
5	C6	H6	0.951
6	C6	C5	1.382(2)
7	C5	H5	0.95
8	C5	C4	1.394(2)
9	C4	H4A	0.95
10	C4	C3	1.394(2)
11	C3	C8	1.400(2)
12	C8	H8	0.949
13	C4	H4A	0.95
14	C3	C2	1.476(2)
15	C2	H2A	0.949
16	C2	N1	1.261(1)
17	N1	C1	1.457(1)
18	N1	C1	1.457(1)

19	H1C	C1	0.98
20	H1A	C1	0.98
21	H1B	C1	0.98

Number	Atom1	Atom2	Atom3	Angle
1	O2	N2	O1	123.29(9)
2	O2	N2	C7	118.75(9)
3	O1	N2	C7	117.96(9)
4	C2	N1	C1	118.1(1)
5	H4A	C4	C3	119.7
6	H4A	C4	C5	119.7
7	C3	C4	C5	120.6(1)
8	H8	C8	C3	120.8
9	H8	C8	C7	120.7
10	C3	C8	C7	118.54(9)
11	N1	C2	H2A	119.2
12	N1	C2	C3	121.6(1)
13	H2A	C2	C3	119.2
14	C4	C3	C8	119.4(1)
15	C4	C3	C2	120.96(9)
16	C8	C3	C2	119.64(9)
17	N2	C7	C8	118.24(9)
18	N2	C7	C6	118.94(9)
19	C8	C7	C6	122.8(1)
20	C7	C6	H6	120.8
21	C7	C6	C5	118.3(1)
22	H6	C6	C5	120.9
23	C4	C5	C6	120.4(1)
24	C4	C5	H5	119.9
25	C6	C5	H5	119.8
26	N1	C1	H1A	109.5
27	N1	C1	H1B	109.4
28	N1	C1	H1C	109.5
29	H1A	C1	H1B	109.5
30	H1A	C1	H1C	109.5
31	H1B	C1	H1C	109.5
32	C10	N3	C9	117.43(9)

33	O3	N4	O4	123.47(9)
34	O3	N4	C15	118.40(9)
35	O4	N4	C15	118.13(9)
36	N4	C15	C16	117.99(9)
37	N4	C15	C14	118.83(9)
38	C16	C15	C14	123.2(1)
39	C12	C11	C16	119.4(1)
40	C12	C11	C10	119.7(1)
41	C16	C11	C10	120.8(1)
42	C11	C12	H12	119.6
43	C11	C12	C13	120.8(1)
44	H12	C12	C13	119.6
45	C15	C16	C11	118.4(1)
46	C15	C16	H16	120.8
47	C11	C16	H16	120.9
48	C15	C14	H14	121
49	C15	C14	C13	117.9(1)
50	H14	C14	C13	121
51	N3	C10	C11	122.6(1)
52	N3	C10	H10	118.7
53	C11	C10	H10	118.7
54	C12	C13	C14	120.3(1)
55	C12	C13	H13	119.8
56	C14	C13	H13	119.9
57	N3	C9	H9A	109.4
58	N3	C9	H9B	109.5
59	N3	C9	H9C	109.5
60	H9A	C9	H9B	109.5
61	H9A	C9	H9C	109.5
62	H9B	C9	H9C	109.5

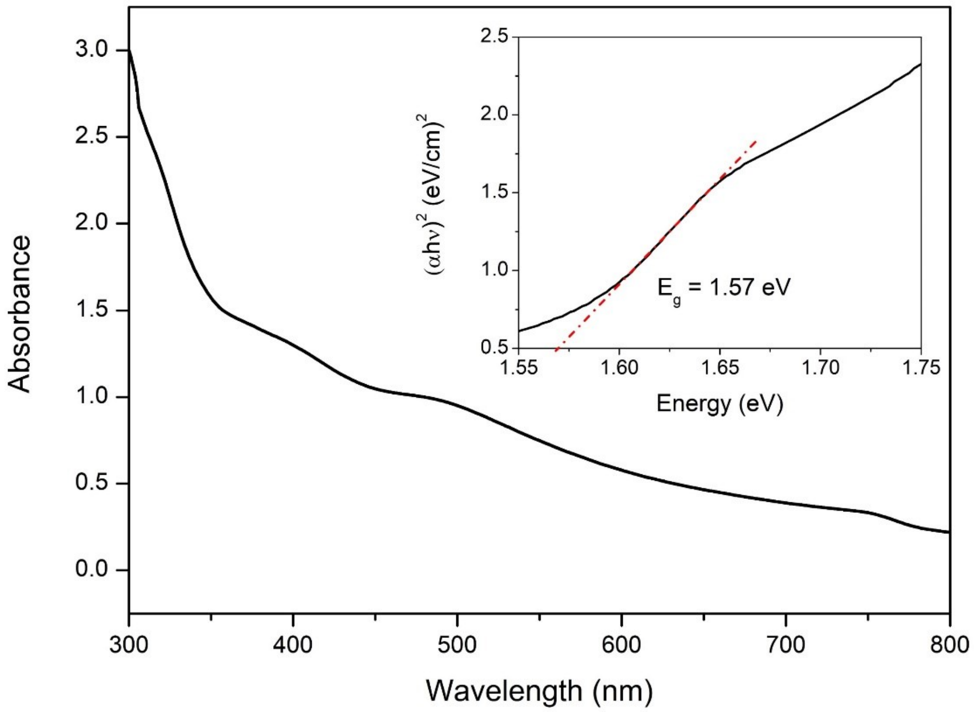

Figure S2. UV-visible spectra of $MAPbI_3$ film grown on a TiO_2/FTO substrate. The inset shows a Tauc plot of optical absorption coefficient versus photon energy for the $MAPbI_3$ film.

Characterization data for imines

(E)-N-benzylidenemethanamine (**Entry 1**).[2]

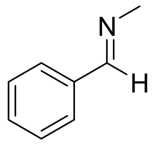

Yield/purity (1.06 g, 98 />99%). Elemental analysis (Found: C, 80.63; H, 7.61; N, 11.75. Calc. For C_8H_9N: C, 80.69; H, 7.54; N, 11.66%). λ_{max}($CHCl_3$)/nm 246 (ε/dm^3 mol^{-1} cm^{-1}; 2.12x10^4). ν_{max}(powder)/cm^{-1} 1650s and 1580w, ν(C=C) and ν(C=N). δ_H (400 MHz; $CDCl_3$; Me_4Si) 3.50 (s, *J* = 1.6 Hz, 3H, N-C-*H*), 7.39 (m, *J* = 1.6 Hz, 1H, C3-*H*, C4-*H*, C5-

H, phenyl), 7.69 (m, *J*= 1.6 Hz, 1H, C2-*H*, C6-*H*, phenyl), 8.25 (s, *J* = 1.6 Hz, 1H, N=C-*H*). δ_C (100 MHz; $CDCl_3$; Me_4Si) 48.2 (N-CH_3), 127.9 (*C*H-3, *C*H-5, phenyl), 128.6 (*C*H-2, *C*H-6, phenyl), 130.5 (*C*H-4, phenyl), 136.2 (*C*H-1, phenyl), 162.6 (N=*C*H) (see Figure S3 and S4). MS (EI+) *m/z* = 118 (100), 119, (54), 120 (5).

(E)-N-(2-methoxybenzylidene)methanamine (**Entry 2**).[3]

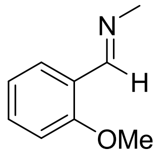

Yield/purity (1.36 g, 98 />99%). Elemental analysis (Found: C, 72.46; H, 7.43; N, 9.39; O, 10.72. Calc. For $C_9H_{11}NO$: C, 72.43; H, 7.34; N, 9.39; O, 11.01 %). $\lambda_{max}(CHCl_3)$/nm 250 (ε/dm^3 mol^{-1} cm^{-1}; 1.65x10^4) and $\lambda_{max}(CHCl_3)$/nm 305 (ε/dm^3 mol^{-1} cm^{-1}; 7.07x10^3). ν_{max}(powder)/cm^{-1} 1642m - 1599m and 1487m - 1464m ν(C=C) and ν(C=N). δ_H (400 MHz; $CDCl_3$; Me_4Si) 3.50 (s, J=1.6 Hz, 3H, N-CH_3), 3.83 (s, 3H, O-CH_3, phenyl), 6.8 (d, *J*= 8.4 Hz, 1H, C3-*H*, phenyl), 6.92 (t, *J*= 8.4 Hz, *J* = 1.6 Hz, 1H, C5-*H*, phenyl), 7.35 (t, *J* = 8.4 Hz, *J* = 1.6 Hz, 1H, C4-*H*, phenyl), 7.9 (d, *J* = 8.4 Hz, *J* = 1.6 Hz, 1H, C6-*H*, phenyl), 8.7 (s, J=1.6 Hz, 1H, N=C-*H*). δ_C (100 MHz; $CDCl_3$; Me_4Si) 48.5 (N-CH_3), 55.4 (N-OCH_3), 110.9 (*C*H-3, phenyl), 120.8 (*C*H-5, phenyl), 124.6 (C_{quart}-1, phenyl), 127.0 (*C*H-6, phenyl), 131.7 (*C*H-4, phenyl), 158.5 (N=*C*H) (see Figure S5 and S6). MS (EI+) *m/z* = 148 (100), 149 (22), 150 (4).

(E)-N-(4-chlorobenzylidene)methanamine (**Entry 3**).[4]

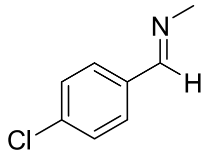

Yield/purity (1.40g, 98/>99%). Elemental analysis (Found: C, 62.55; H, 5.25; Cl, 23.08; N, 9.12. Calc. For C_8H_8ClN: C, 62.58; H, 5.25; Cl, 22.96; N, 9.21 %). λ_{max}($CHCl_3$)/nm 254 (ε/dm^3 mol^{-1} cm^{-1}; 2.91x10^4). ν_{max}(powder)/cm^{-1} 1650m - 1596m and 1488m - 1455m , ν(C=C) and ν(C=N). δ_H (400 MHz; $CDCl_3$; Me_4Si) 3.50 (s, *J* = 1.6 Hz, 3H, N-C*H*$_3$), 7.37 (d, *J* = 8.4 Hz, 2H, C3-*H* and C5-*H*, phenyl), 7.63 (d, *J* = 8.4 Hz, 2H, C2-*H* and C6-*H*, phenyl), 8.21 (s, *J* = 1.6 Hz, 1H, N=C-*H*). δ_C (100 MHz; $CDCl_3$; Me_4Si) 48.2 (N-*C*H$_3$), 128.9 (*C*H-3 and *C*H-5, phenyl), 129.1 (*C*H-2 and *C*H-6, phenyl), 134.7 (C_{quart}-1, phenyl), 136.2 (C_{quart}-4, phenyl), 161.2 (N=*C*H-Ph) (see Figure S7 and S8). MS (EI+) *m/z* = 152 (100), 153 (55), 154 (38), 155 (18), 157 (3).

(E)-N-(4-bromobenzylidene)methanamine (**Entry 4**).[5]

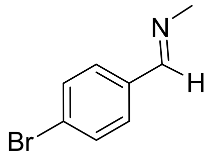

Yield/purity (1.29g, 98/>99%). Elemental analysis (Found: C, 48.51; H, 4.07; N, 7.07; Br, 40.34. Calc. For C_8H_8BrN; C, 48.51; H, 4.07; N, 7.06; Br, 40.24 %). λ_{max}($CHCl_3$)/nm 256 (ε/dm^3 mol^{-1} cm^{-1}; 2.36x10^4). ν_{max}(powder)/cm^{-1} 1650s - 1588s and 1487m - 1453m, ν(C=C) and ν(C=N). δ_H (400 MHz; $CDCl_3$; Me_4Si) 3.49 (s, *J* = 1.6 Hz, 3H, N-C*H*$_3$), 7.52 (d, *J* = 8.4 Hz, 2H, C2-*H* and C6-*H*, phenyl), 7.56 (d, *J* = 8.4 Hz, 2H, C3-*H* and C5-*H*, phenyl), 8.21 (s, *J* = 1.6 Hz, 1H, N=C-*H*). δ_C (100 MHz; $CDCl_3$; Me_4Si) 48.3 (N-*C*H$_3$), 124.9 (C_{quart}-4, phenyl), 129.4 (*C*H-2 and *C*H-6, phenyl), 131.9 (*C*H-3 and *C*H-5, phenyl), 135.2 (C_{quart}-1, phenyl), 161.3 (N=*C*H-Ph) (see Figure S9 and S10). MS (EI+) *m/z* = 196 (100), 197 (57), 198 (100), 199 (54), 200 (4).

(E)-N-(3-nitrobenzylidene)methanamine (**Entry 5**).[6]

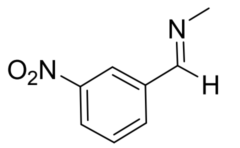

Yield/purity (1.51 g, 97/>99%). mp 54.7 °C – 55 °C (CH_2Cl_2/hexane) lit. 55.1-55.8 °C. Elemental analysis (Found: C, 58.53; H, 4.91; N, 17.06; O, 19.49. Calc. For $C_8H_8N_2O_2$: C, 58.54; H, 4.92; N, 17.05; O, 19.48 %). λ_{max}($CHCl_3$)/nm 241 (ε/dm^3 mol^{-1} cm^{-1}; 2.53x10^4). ν_{max}(powder)/cm^{-1} 1648m and 1520s, ν(C=C) and ν(C=N). δ_H (400 MHz; $CDCl_3$; Me_4Si) 3.57 (s, J=1.6 Hz, 3H, N-C*H*$_3$), 7.58 (t, *J*= 8.0 Hz, 1H, C5-*H*, phenyl), 8.04 (d, *J* = 8.0 Hz, 1H, C4-*H*, phenyl), 8.24 (d, *J*= 8.0 Hz, 1H, C6-*H*, phenyl), 8.36 (s, *J*= 1.6 Hz, 1H, C2-*H*, phenyl), 8.53 (s, J=1.6 Hz, 1H, N=C-*H*). δ_C (100 MHz; $CDCl_3$; Me_4Si) 48.2 (N-*C*H$_3$), 122.5 (*C*H-2, phenyl) 124.9 (*C*H-4, phenyl), 129.6 (*C*H-5, phenyl), 133.4 (*C*H-6, phenyl), 137.9 (C_{quart}-1, phenyl), 148.5 (*C*-NO_2), 159.9 (N=*C*H-Ph). (see Figure S11 and S12). MS (EI+) *m/z* = 163 (100), 164 (75), 165 (7).

2-[*(E)*-(methylimino)methyl)]phenol (Entry 6).[7]

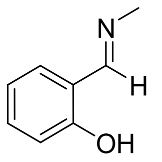

Yield/purity (1.20 g, 98/>99%). Elemental analysis (Found: C, 71.09; H, 6.71; N, 10.36; O, 11.84. Calc. For C_8H_9NO: C, 71.14; H, 6.68; N, 10.41; O, 11.76%). λ_{max}($CHCl_3$)/nm 255 (ε/dm^3 mol^{-1} cm^{-1}; 9.0x10^3) and λ_{max}($CHCl_3$)/nm 315 (ε/dm^3 mol^{-1} cm^{-1}; 3.47x10^3). ν_{max}(powder)/cm^{-1} 1635s - 1583m and 1495m – 1451m, ν(C=C) and ν(C=N). 3.45 (s, J=1.6 Hz, 3H, N-C*H*$_3$), 6.85 (t, *J*=7.6 Hz, 1H, C5-*H,* phenyl), 6.96 (d, *J* = 8.0 Hz, 1H, C3-*H*, phenyl), 7.21 (d, *J* = 7.6 Hz, 1H, C6-*H*, phenyl), 7.27 (t, *J* = 8.0 Hz, 1H, C4-*H*, phenyl), 8.30 (s, J=1.6 Hz, 1H, N=C-*H*). 13.5 (s, -OH, exchangeable signal) δ_C (100 MHz;

CDCl$_3$; Me$_4$Si) 46.0 (N-*C*H$_3$), 117.0 (*C*H-3, phenyl) 118.5 (*C*H-5, phenyl), 118.9 (*C*H-1, phenyl), 131.1 (*C*H-6, phenyl), 132.1 (*C*H-4, phenyl), 161.2 (*C*-OH, phenyl), 166.2 (N=*C*H-Ph). (see Figure S13 and S14). MS (EI+) *m/z* = 134 (89), 135 (100), 136 (9).

***(E)-N-((E)*-3-phenylallylidene)methanamine (Entry 7).**

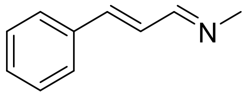

Yield/purity (1.32 g, 98/>99%). Elemental analysis (Found: C, 82.72; H, 7.64; N, 9.65. Calc. For $C_{10}H_{11}N$: C, 82.66; H, 7.72; N, 9.65 %). λ_{max}(CHCl$_3$)/nm 281 (ε/dm^3 mol^{-1} cm^{-1}; 2.67x10^4). ν_{max}(powder)/cm^{-1} 1637s and 1448m, ν(C=C) and ν(C=N). δ_H (500 MHz; CDCl$_3$; Me$_4$Si) 3.41 (s, 3H, N-C-*H*), 6.87 (dd, *J* = 16 Hz, 1H, Ph-CH=CH_b-CH=N-CH$_3$), 6.92 (d, *J* = 16 Hz, 1H, Ph-CH_c=CH-CH=N-CH$_3$), 7.30 (t, *J* = 7.5 Hz, 1H, C4-*H*, phenyl), 7.35 (t, *J* = 7.5 Hz, 2H, C3-*H* and C5-*H*, phenyl), 7.46 (d, *J* = 7.5 Hz, 2H, 2H, C2-*H* and C6-*H*, phenyl), 8.0 (dd, *J* = 7.5 Hz and *J* = 1.5 Hz, 1H, Ph-CH=CH-CH_a=N-CH$_3$). δ_C (100 MHz; CDCl$_3$; Me$_4$Si) 48.2 (N-*C*H$_3$), 127.1 (Ph-CH=*C*H$_b$-CH=N-CH$_3$, phenyl), 128.1 (*C*H-2 and *C*H-6, phenyl), 128.7 (*C*H-3 and *C*H-5, phenyl), 129.0 (*C*H-4, phenyl), 135.7 (*C*H-1, phenyl), 141.0 (Ph-*C*H$_c$=CH-CH=N-CH$_3$), 164.0 (Ph-CH=CH-*C*H$_a$=N-CH$_3$). (see Figure S15 and S16). MS (EI+) *m/z* = 144 (100), 145 (22), 146 (2).

***(E)-N-((E)*-3-(2-methoxyphenyl)allylidene)methanamine (Entry 8).**

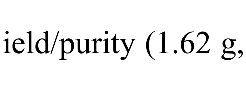

ield/purity (1.62 g, 98/>99%). Elemental analysis (Found: C, 75.40; H, 7.48; N, 7.99; O, 9.13. Calc. For $C_{11}H_{13}NO$: C, 75.49; H, 7.46; N, 7.99; O, 9.04 %). λ_{max}(CHCl$_3$)/nm 277

(ε/dm^3 mol^{-1} cm^{-1}; 2.19x10^4) and λ_{max}($CHCl_3$)/nm 318 (ε/dm^3 mol^{-1} cm^{-1}; 1.32x10^4). ν_{max}(powder)/cm^{-1} 1634m and 1487m, ν(C=C) and ν(C=N). δ_H (500 MHz; $CDCl_3$; Me_4Si) 3.40 (s, 3H, N-C-*H*), 3.85 (s, 3H, C2-OCH_3, phenyl), 6.89 (t, *J* = 7.5 Hz, 1H, C5-*H*, phenyl), 6.91 (dd, *J* = 16 Hz, 1H, Ph-CH=CH_b-CH=N-CH_3), 6.94 (t, *J* = 7.5 Hz, 1H, C4-*H*, phenyl), 7.27 (d, *J* = 16 Hz, 1H, Ph-CH_c=CH-CH=N-CH_3), 7.29 (d, *J* = 7.5 Hz, 1H, C3-*H*, phenyl), 7.50 (d, *J* = 7.5 Hz, 1H, C3-*H*, phenyl), 8.02 (dd, *J* = 7.5 Hz and *J* = 1.5 Hz, 1H, Ph-CH=CH-CH_a=N-CH_3), δ_C (100 MHz; $CDCl_3$; Me_4Si) 48.1 (N-*C*H_3), 55.4 (C2-O*C*H_3, phenyl), 110.9 (*C*H-3, phenyl), 120.7 (Ph-CH=*C*H_b-CH=N-CH_3, phenyl), 124.7 (*C*H-5, phenyl), 127.4 (*C*H-1, phenyl), 128.7 (*C*H-4, phenyl), 130.2 (*C*H-6, phenyl), 136.2 (Ph-*C*H_c=CH-CH=N-CH_3), 157.2 (*C*H-2, phenyl), 164.9 (Ph-CH=CH-*C*H_a=N-CH_3). (see Figure S17 and S18). MS (EI+) *m/z* = 174 (100), 175 (35), 176 (5).

Figure S3 ^{1}H NMR Spectrum of **Entry 1**

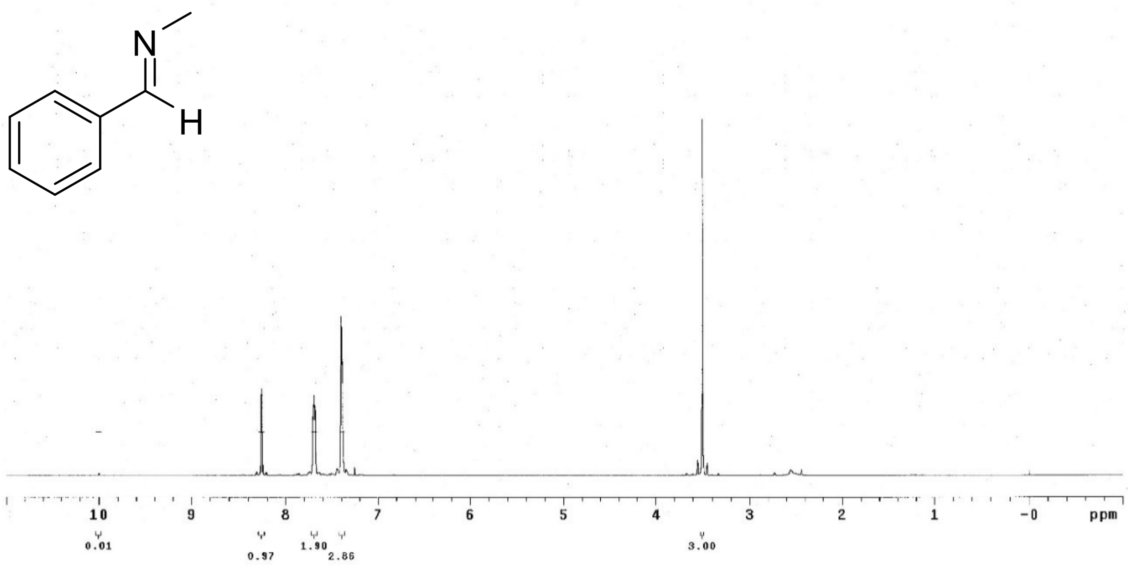

Figure S4 ^{13}C NMR Spectrum of **Entry 1**

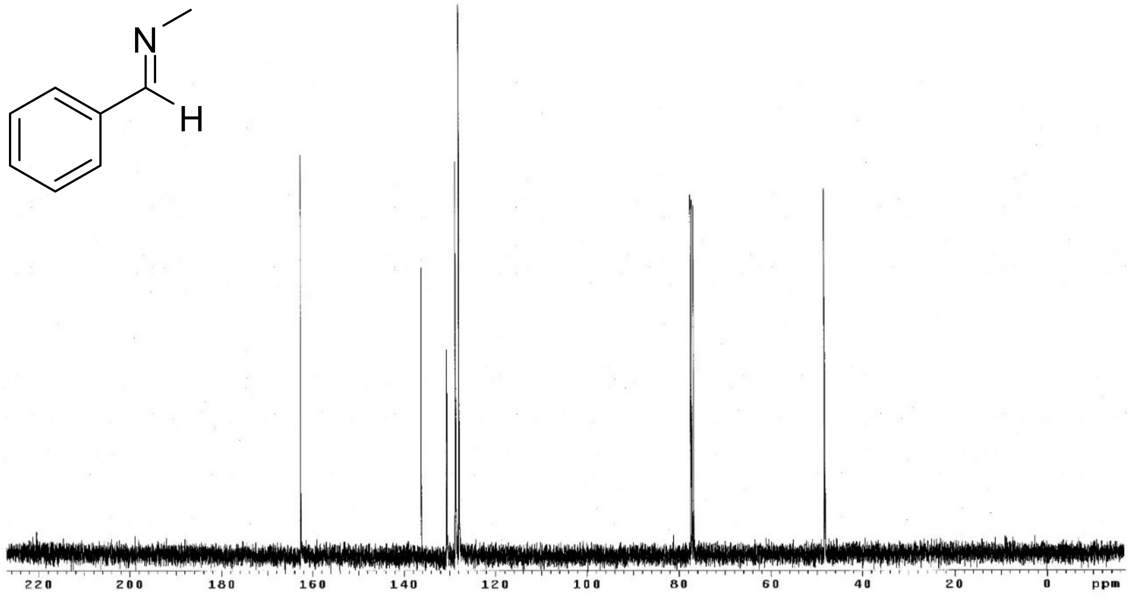

Figure S5 ^{1}H NMR Spectrum of **Entry 2**

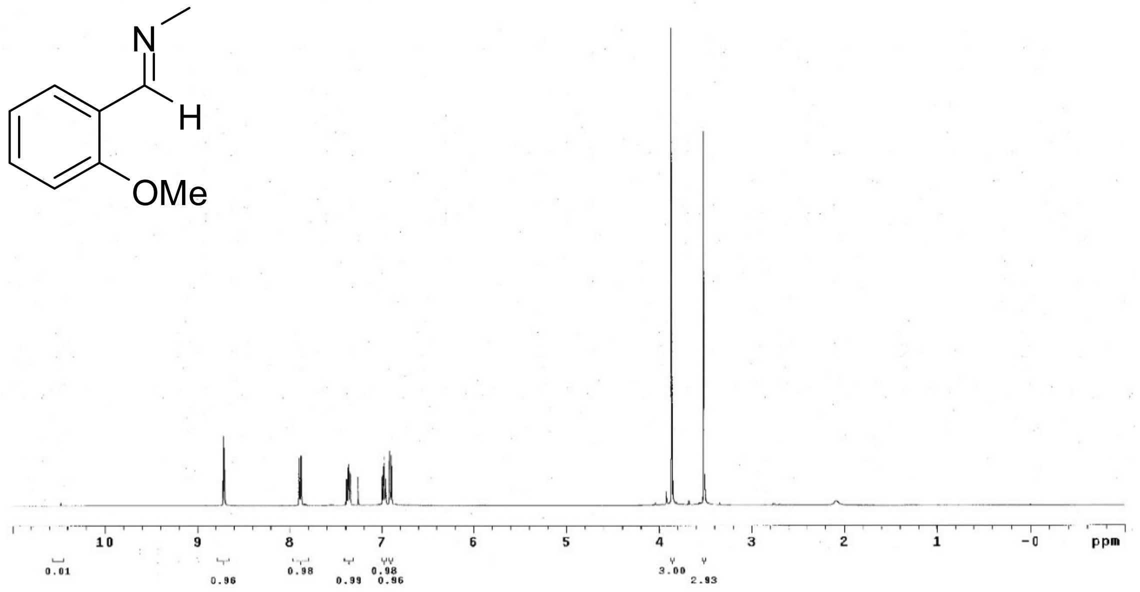

Figure S6 ^{13}C NMR Spectrum of **Entry 2**

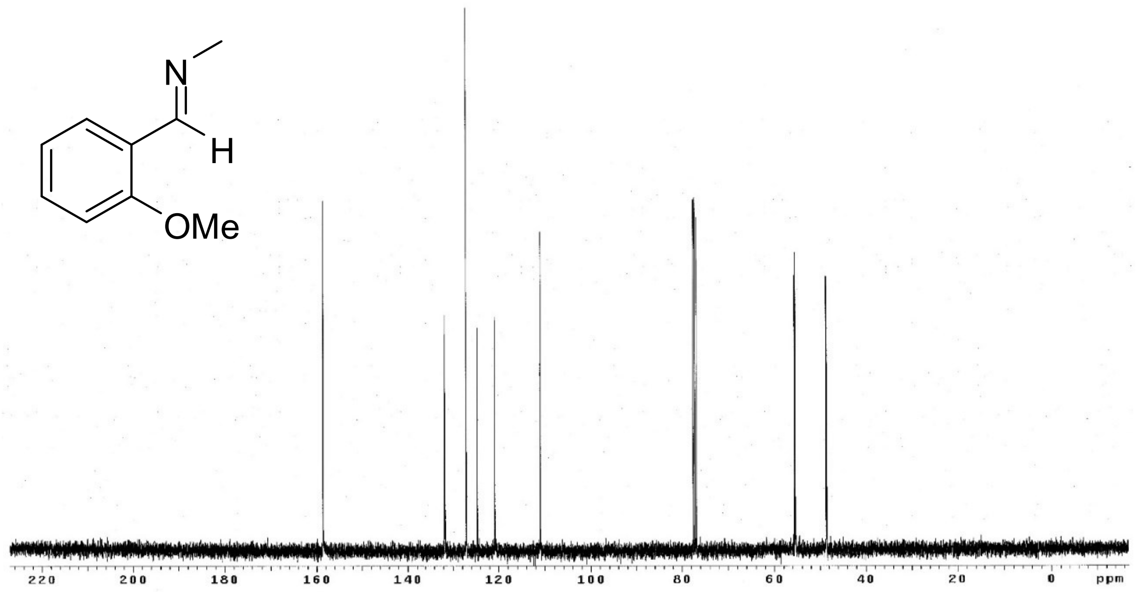

Figure S7 ^{1}H NMR Spectrum of **Entry 3**

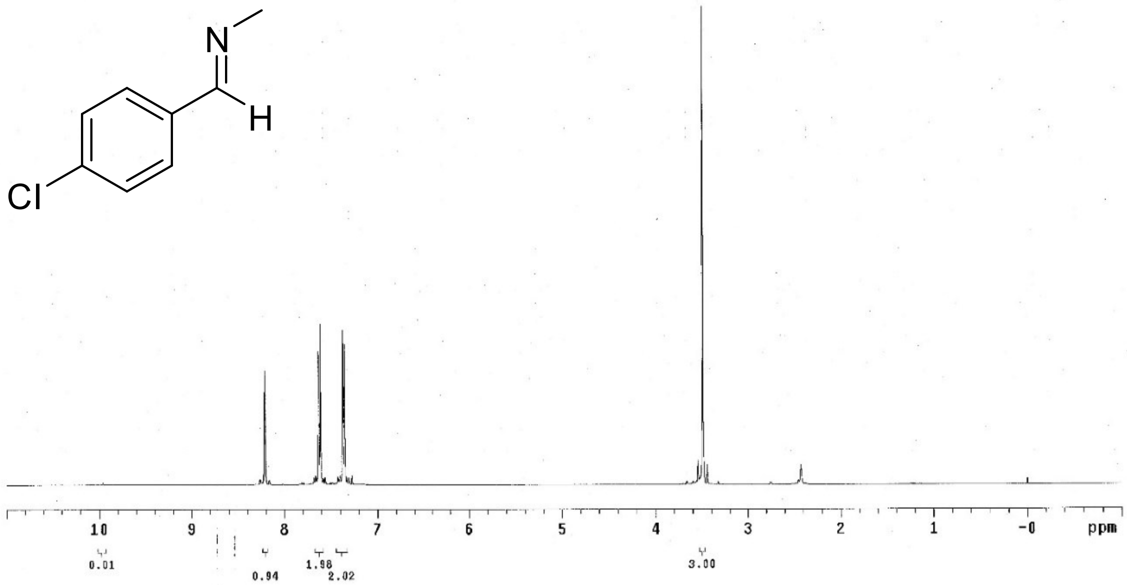

Figure S8 ^{13}C NMR Spectrum of **Entry 3**

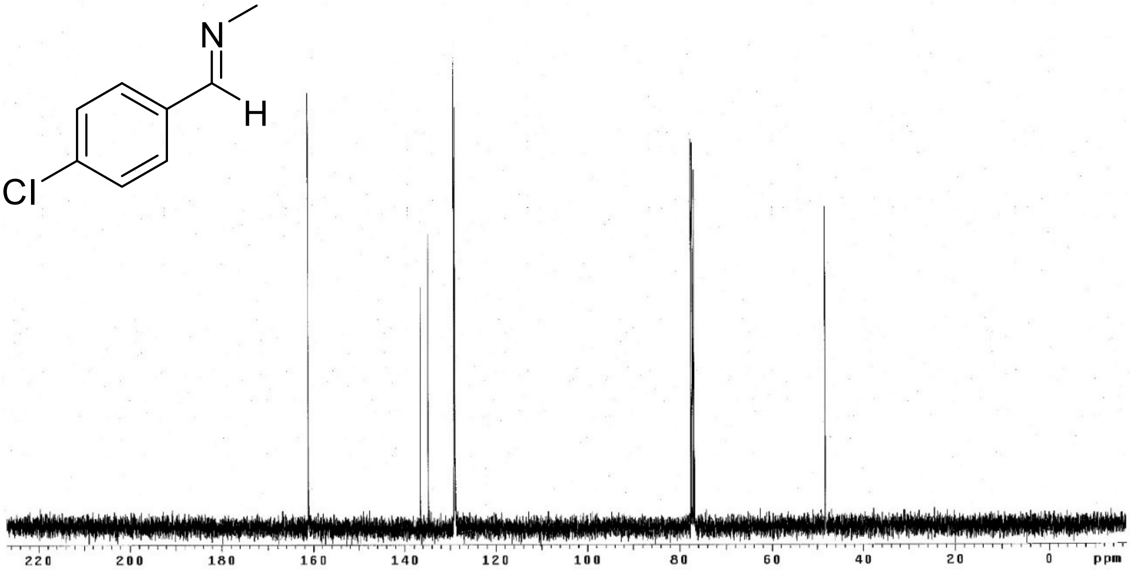

Figure S9 ^{1}H NMR Spectrum of **Entry 4**

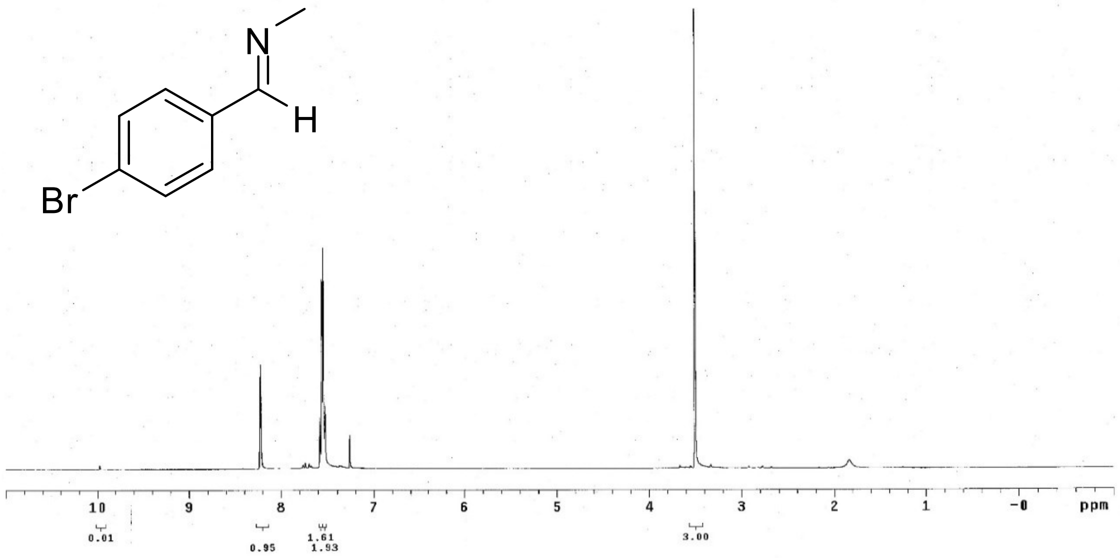

Figure S10 ^{13}C NMR Spectrum of **Entry 4**

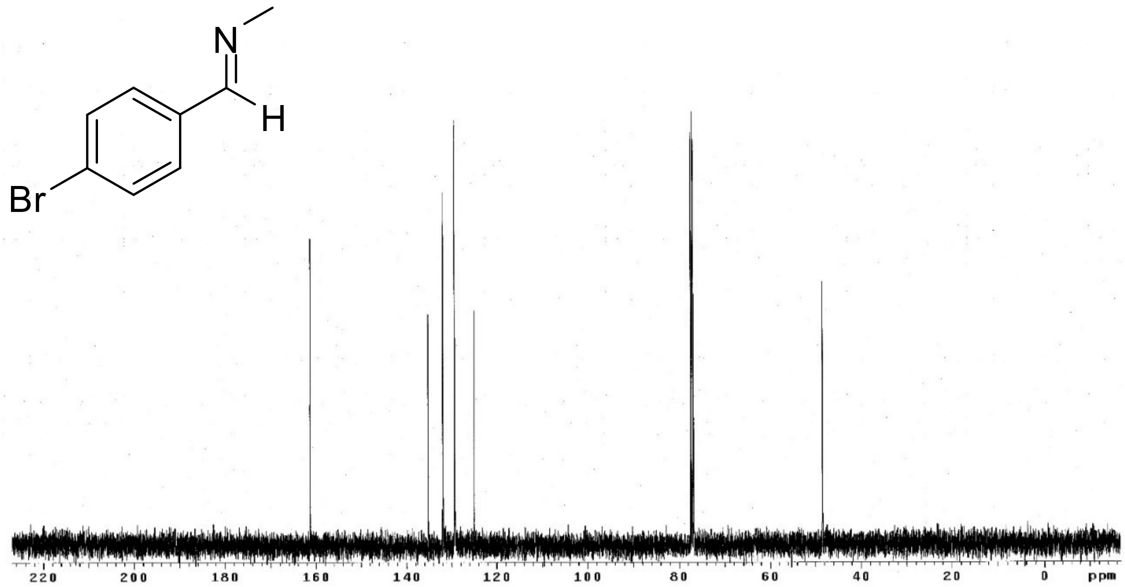

Figure S11 ^{1}H NMR Spectrum of **Entry 5**

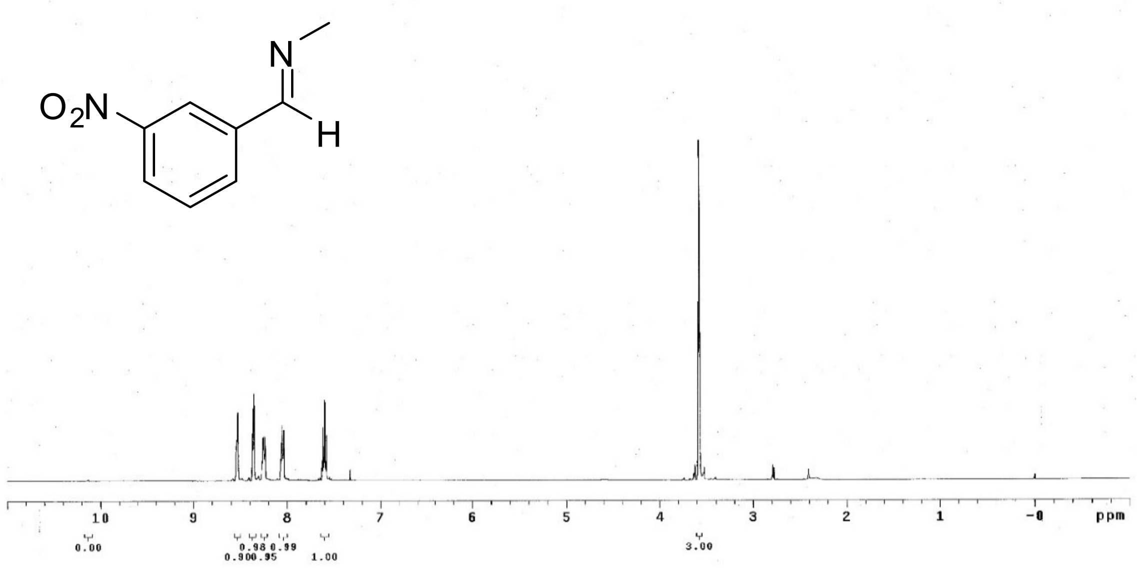

Figure S12 ^{13}C NMR Spectrum of **Entry 5**

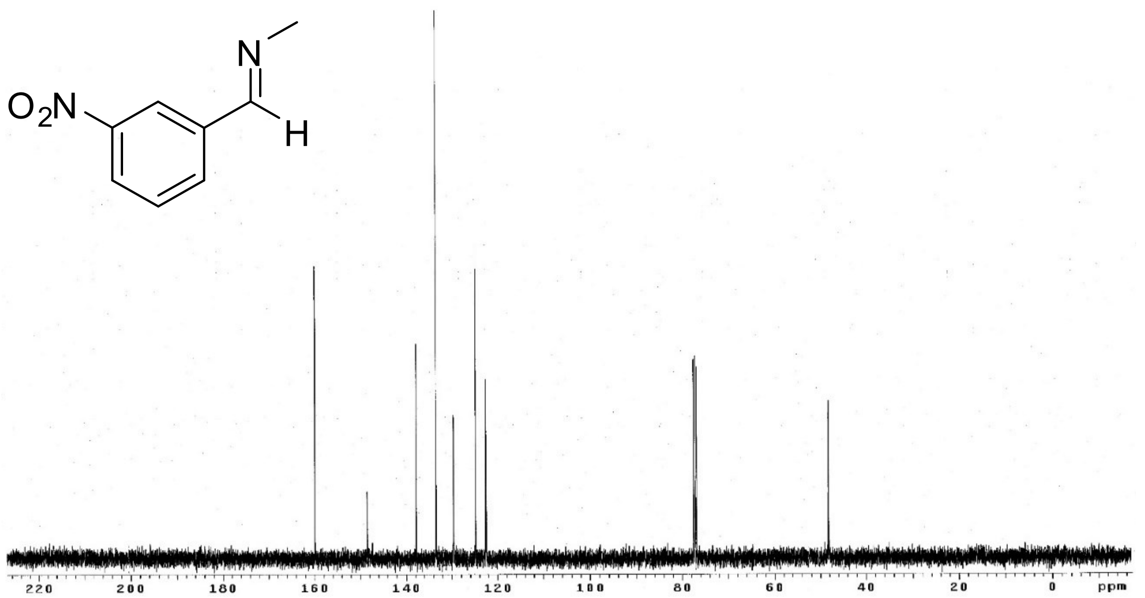

Figure S13 ^{1}H NMR Spectrum of **Entry 6**

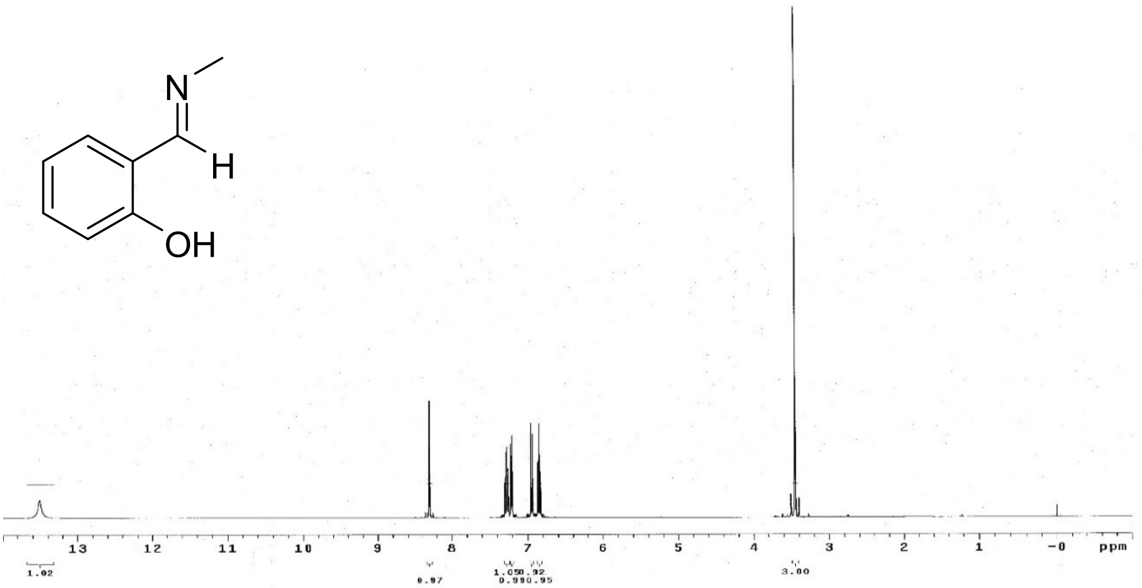

Figure S14 ^{13}C NMR Spectrum of **Entry 6**

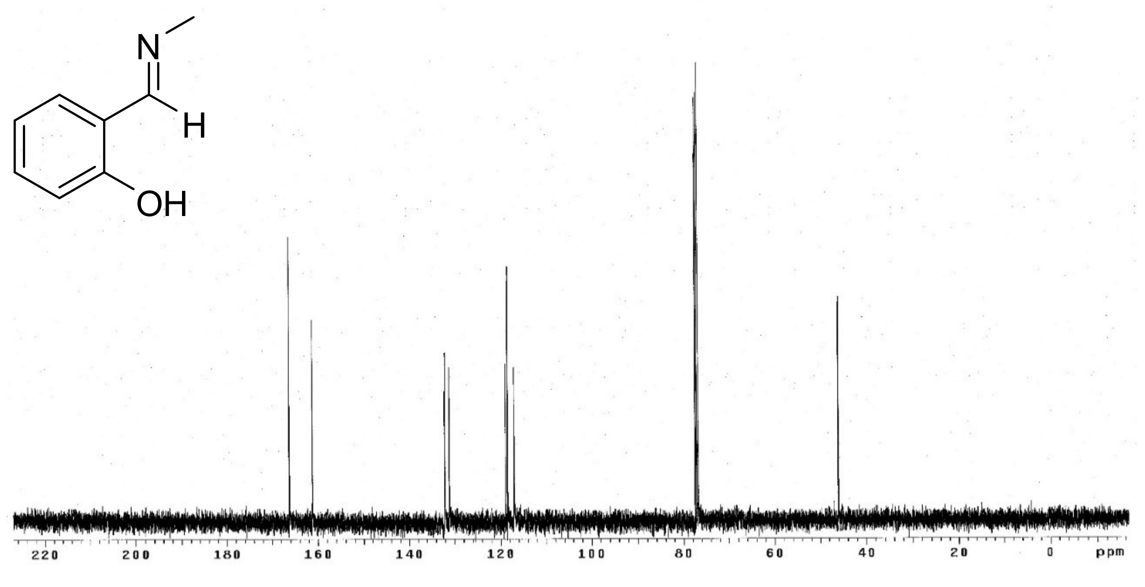

Figure S15 ^{1}H NMR Spectrum of **Entry 7**

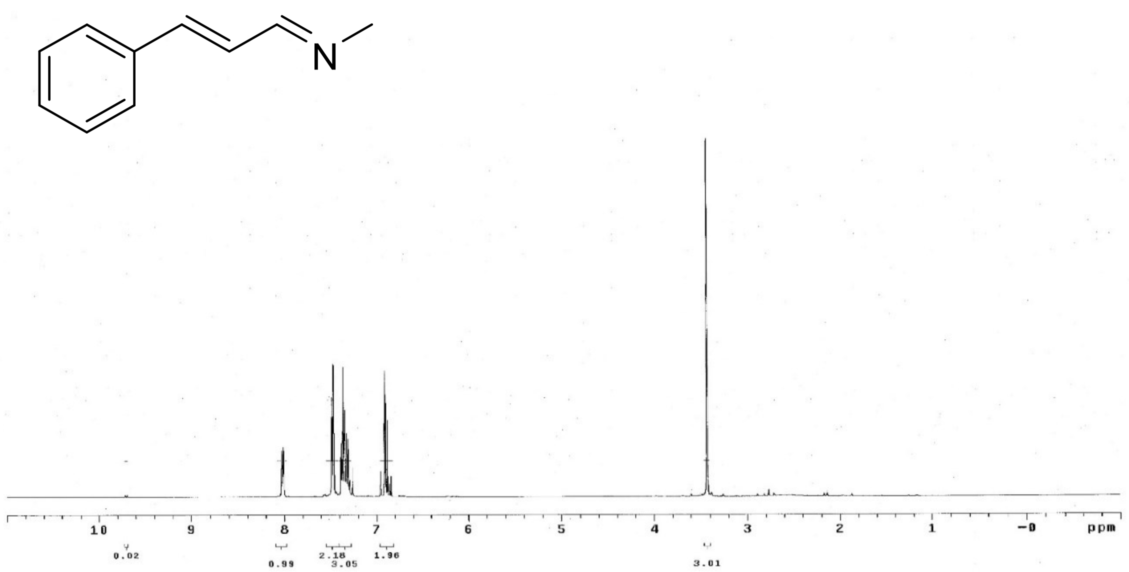

Figure S16 ^{13}C NMR Spectrum of **Entry 7**

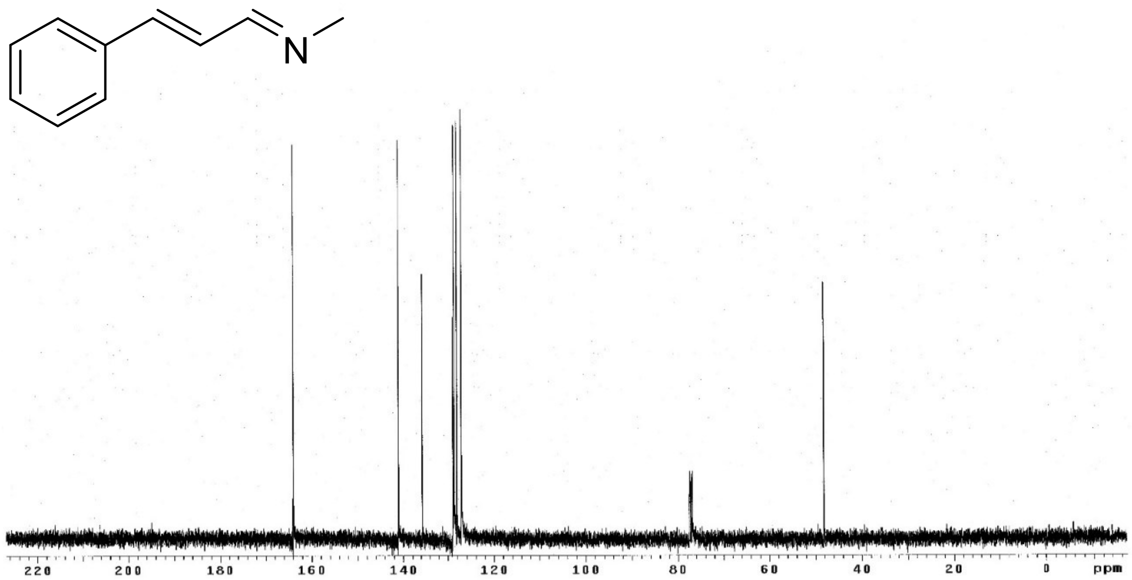

Figure S17 ^{1}H NMR Spectrum of **Entry 8**

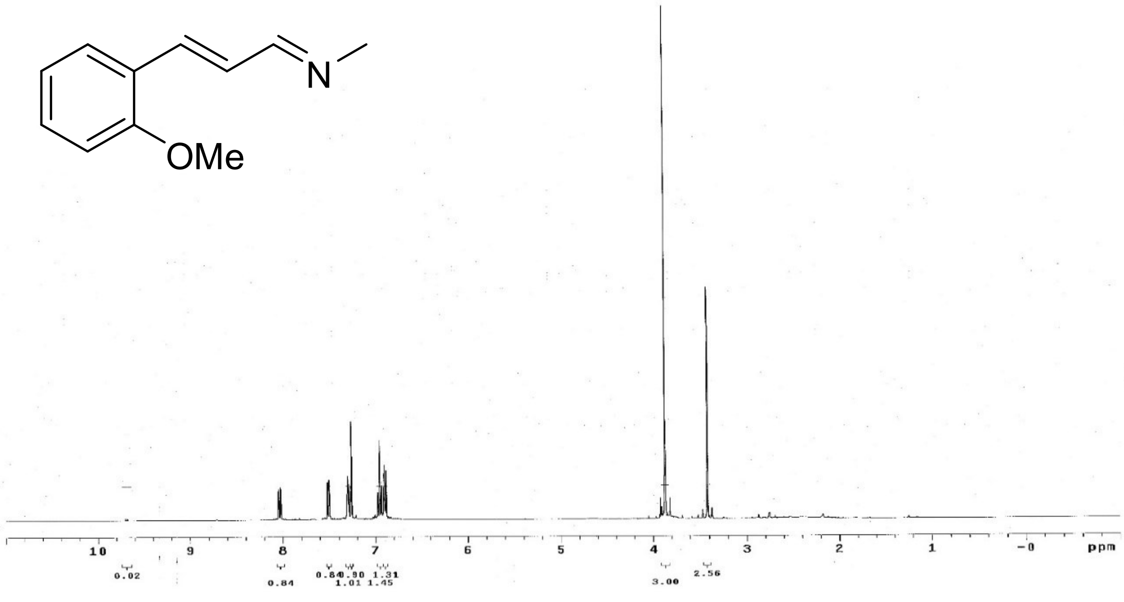

Figure S18 ^{13}C NMR Spectrum of **Entry 8**

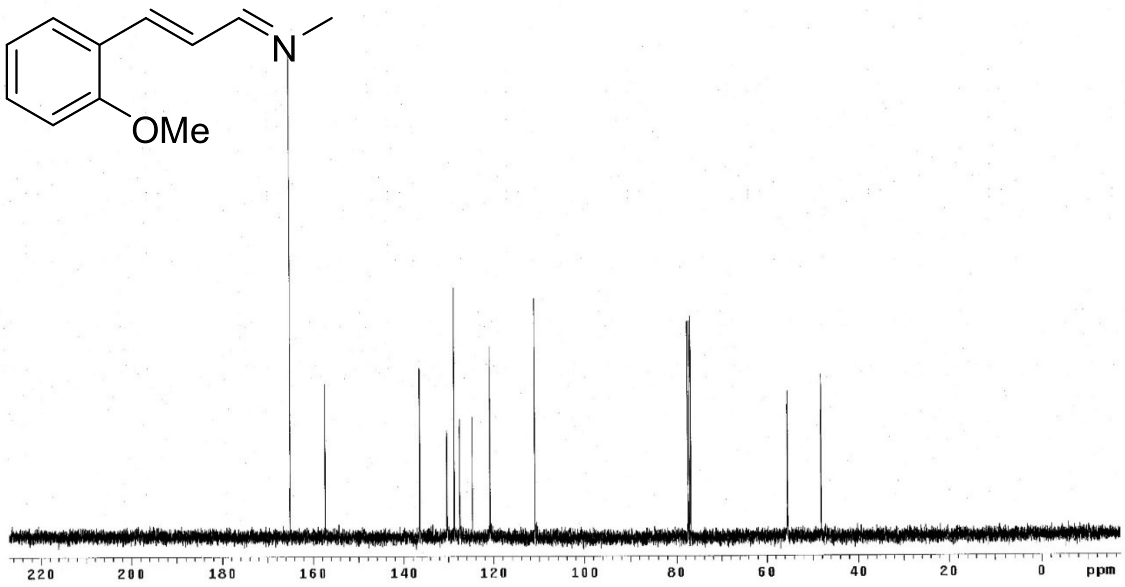

Figure S19 ¹H NMR Spectrum of **MMA solution** (33wt% in EtOH) and **trans-cinnamaldehyde**.

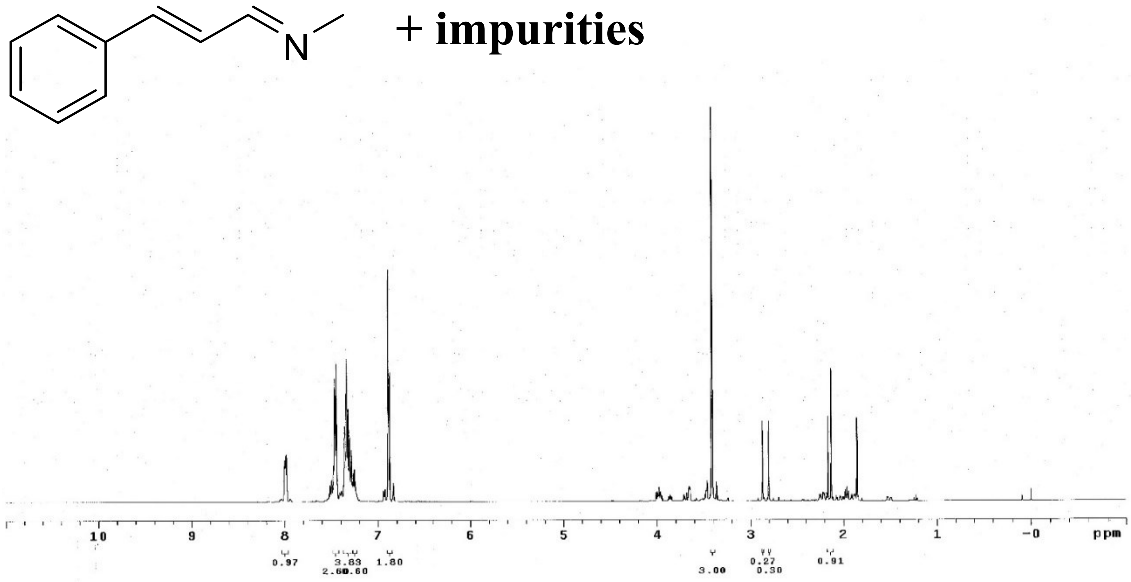

Figure S20 ¹³C NMR Spectrum of **MMA solution** (33wt% in EtOH) and **trans-cinnamaldehyde**.

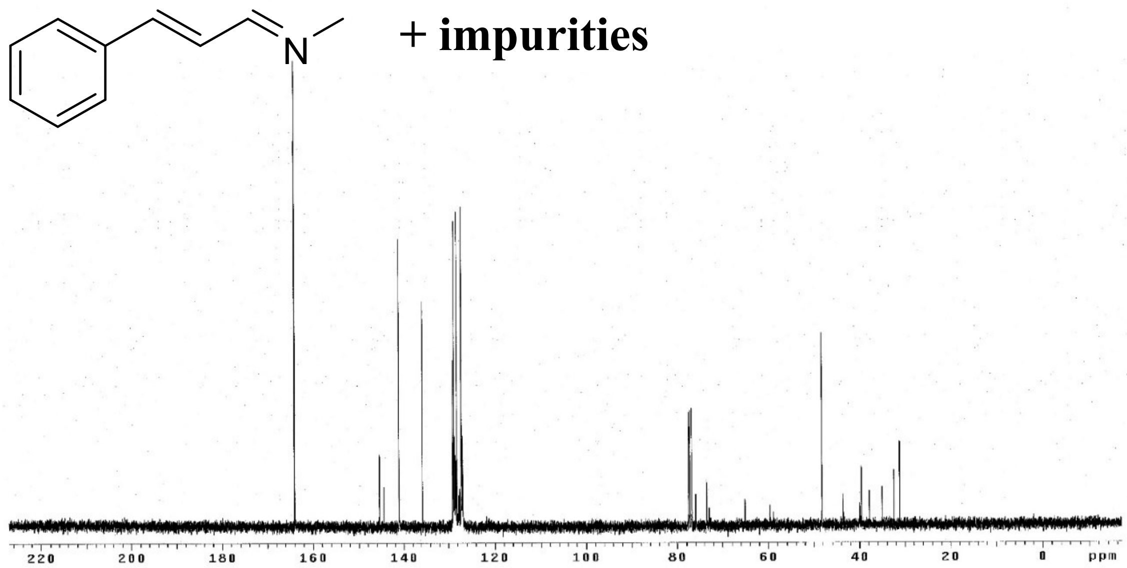

References

1. G. M. Sheldrick, *SHELXTL/PC Version 6.12 for Windows XP*, Bruker AXS Inc., Madison, WI, 2001.
2. W. Schilf / Journal of Molecular Structure 689 (**2004**) 245–249, L. Paquin, J. Hamelin, F. Texier-Boullet, *Synthesis*, **2006**, 1652-1656, Compare to a commercial product: CAS number 622-29-7.
3. L. Piazzi et al. / Bioorg. Med. Chem. 15 (**2007**) 575–585. Compare to a commercial product: CAS number. 1125-90-2.
4. L. Paquin, J. Hamelin, F. Texier-Boullet, *Synthesis*, **2006**, 1652-1656. L. Piazzi et al. / Bioorg. Med. Chem. 15 (**2007**) 575–585., Jennings, W. B.; Wilson, V. E.; Boyd, D. R.; Coulter, P. B., *Organic Magnetic Resonance* **1983,** *21* (4), 279-286. Compare to a commercial product: CAS number. 13114-22-2.
5. The Journal of Organic Chemistry **2002,** 67 (23), 8191-8196. Jennings, W. B.; Wilson, V. E.; Boyd, D. R.; Coulter, P. B., *Organic Magnetic Resonance* **1983,** *21* (4), 279-286.
6. Connolly, T. J.; Constantinescu, A.; Lane, T. S.; Matchett, M.; McGarry, P.; Paperna, M., *Organic Process Research & Development* **2005,** *9* (6), 837-842. Compare to a commercial product: CAS number. 121004-44-2.
7. W. Schilf / Journal of Molecular Structure 689 (2004) 245–249, *Russian Journal of Coordination Chemistry* **2009,** *35* (2), 120-127. Compare to a commercial product: CAS number. 107910-69-0.
8. Hauser, C. R.; Manyik, R. M.; Brasen, W. R.; Bayless, P. L., The Journal of Organic Chemistry **1955,** 20 (8), 1119-1128. Winn, M.; Dunnigan, D. A.; Zaugg, H. E., The Journal of Organic Chemistry **1968**, 33 (6), 2388-2392., Jennings, W. B.; Wilson, V. E.; Boyd, D. R.; Coulter, P. B., *Organic Magnetic Resonance* **1983,** *21* (4), 279-286. Compare to a commercial product: CAS number. 13280-16-5.
